# Information Fusion Based on Complementary Filter for SINS/CNS/GPS Integrated Navigation System of Aerospace Plane

**DOI:** 10.3390/s20247193

**Published:** 2020-12-15

**Authors:** Yanming Zhao, Gongmin Yan, Yongyuan Qin, Qiangwen Fu

**Affiliations:** School of Automation, Northwestern Polytechnical University, Xi’an 710129, China; zhaoyanming@mail.nwpu.edu.cn (Y.Z.); qinyongyuan@nwpu.edu.cn (Y.Q.); fuqiangwen@nwpu.edu.cn (Q.F.)

**Keywords:** aerospace plane, integrated navigation, complementary filter, information fusion

## Abstract

In order to solve the problems of heavy computational load and poor real time of the information fusion method based on the federated Kalman filter (FKF), a novel information fusion method based on the complementary filter is proposed for strapdown inertial navigation (SINS)/celestial navigation system (CNS)/global positioning system (GPS) integrated navigation system of an aerospace plane. The complementary filters are designed to achieve the estimations of attitude, velocity, and position in the SINS/CNS/GPS integrated navigation system, respectively. The simulation results show that the proposed information fusion method can effectively realize SINS/CNS/GPS information fusion. Compared with FKF, the method based on complementary filter (CF) has the advantages of simplicity, small calculation, good real-time performance, good stability, no need for initial alignment, fast convergence, etc. Furthermore, the computational efficiency of CF is increased by 94.81%. Finally, the superiority of the proposed CF-based method is verified by both the semi-physical simulation and real-time system experiment.

## 1. Introduction

The aerospace plane is a reusable aerospace vehicle with the features of aircraft, spacecraft, and carrier, in which the accuracy and reliability requirements of the navigation system are very high due to its wide flight envelope, complex flight environment, and special mission [1]. The common navigation systems in aerospace vehicle include the strapdown inertial navigation system (SINS), global positioning system (GPS), celestial navigation system (CNS), and so on. Considering the black-out phenomenon in atmospheric reentry, the single SINS navigation using single-axis rotation modulation [2] or a second-order damping scheme [3] was adopted for the navigation of hypersonic vehicles. In order to serve the needs of spacecraft, the global navigation satellite system (GNSS) based on GPS [4], global navigation satellite system (GLONASS) [5], GPS/Galileo [6], or GPS/beidou navigation satellite system (BDS) [7] was proposed. An approach using the measurements from GNSS was proposed for the navigation of circumlunar spacecraft [8] or high-Earth-orbits spacecraft [9]. References [10,11] studied the high-precision autonomous celestial navigation based on astronomical information for spacecraft.

In view of the advantages and disadvantages of each navigation subsystem, it is difficult for any single navigation system to provide high-precision navigation parameters independently. In this background, the method of integrated navigation was proposed. Compared with a single navigation system, the integrated navigation system has higher accuracy as well as better reliability and information completeness; therefore, it can be applied for the scenario of an aerospace plane. In [12], a GPS/SINS integrated navigation algorithm in the launch-centered Earth-fixed (LCEF) frame was proposed to suit the features of hypersonic vehicles and meet the requirements of their flight control system. For the navigation applications of a hypersonic vehicle, the design of a tightly coupled SINS/GNSS integrated navigation system was presented to resist the disturbance of measurement errors, which adopted the innovation orthogonality-based robust unscented Kalman filter (IO-RUKF) [13] or order-reduction robust filter [14]. Reference [15] reviewed the inertial navigation system (INS)/CNS integrated navigation technology. For space vehicles of probes and hypersonic cruise vehicles, two tightly coupled INS/CNS integrated navigation methods were proposed, which adopted the accurate CNS celestial measurements assisted by the corrected infrared Earth sensor [16] and the weighted multi-stars observations according to the different error levels of starlight observations [17], respectively. Considering the high navigation requirements and complex practical environment of high dynamic aircraft, the INS/CNS/GPS integrated navigation approach was presented, which used the robust filtering algorithm based on extended H∞ filter [18], Kalman filter assisted by neural network ensembles [19], or unscented Kalman filter (UKF)-based Federated filter [20]. In [21], a new INS/GNSS/CNS integrated navigation method for hypersonic cruising aircrafts (HCVs) based on non-Keplerian orbits mode was presented. For autonomous SINS/GPS/star sensor (SS) navigation of space vehicle, the information fusion algorithms of the extend Kalman filter [22] and federated Kalman filter [23] were designed, respectively. In [24], a federated Sage–Husa adaptive filter was proposed for a SINS/CNS/GNSS navigation system with time-varying noise. For INS/GNSS/CNS integration, Hu et al. proposed two novel information fusion methods: the modified federated Kalman filter (MFKF) based on state decomposition [25] and the matrix weighted multi-sensor data fusion methodology with a two-level structure [26]. Gao et al. presented two multi-sensor optimal data fusion methods with “bottom–top-levels” structures for INS/GNSS/CNS integrated navigation, of which one was based on a UKF filter [27], and another was based on the adaptive fading unscented Kalman filter [28]. However, the data fusion methods for INS/CNS/GPS integrated navigation in the existing literature are mostly implemented by Kalman filter (KF) or federated Kalman filter (FKF). Since the state dimension of FKF in an INS/CNS/GPS integrated navigation system is large, the computational load is heavy and the computational efficiency is low, and the precise filtering model and parameters are required for FKF. In the case of actual non-Gaussian noise or a large model error, the filtering divergence may occur. State observer is also a method of state estimation for a nonlinear system (particularly speed estimation), such as the approximately-reconstruct-state-variables observer [29], approximated-velocity-switching observer [30], designed-for-Lipschitz-nonlinear-system observer [31], etc. However, the estimation method based on state observer has the following disadvantages: (1) It is difficult to select the feedback gain matrix of the general state observer, which needs to be determined according to experiments or experience; (2) Without consideration of the random error of the system, the estimation accuracy of the observer is affected by the stochastic noise of the system; (3) The design of the state observer requires the system to be completely observable, but the integrated navigation system generally does not meet the requirement of complete observability. Therefore, the estimation method based on the state observer is not suitable for a complex multi-source integrated navigation system.

Compared with FKF, the design of complementary filter (CF) is based on the frequency-domain characteristics of navigation errors of each subsystem, and it does not require the exact system model and noise statistics. The CF filter has the advantages of a simple algorithm, low computational load, good real-time computations, etc. When it is used in an integrated navigation system, CF can achieve the closely same filtering precision as FKF [32,33,34]. In this paper, a novel information fusion method based on complementary filter is proposed for an SINS/CNS/GPS integrated navigation system to improve both the computational efficiency and real-time performance. By utilizing the method of CF filtering, the attitude/speed/position estimations of a SINS/CNS/GPS integrated navigation system are realized, respectively. In addition, the proposed information fusion method has the advantages of low computation load, good stability, and steady-state accuracy, which meet the requirements of an integrated navigation system for an aerospace plane.

The rest of this paper is organized as follows: Section 2 introduces the coordinate frames in use, inertial navigation mechanization, and CNS measurement transformation from the i frame to the n frame. Section 3 introduces the principle of complementary filter and designs the information fusion algorithms for an SINS/CNS/GPS integrated navigation system. Section 4 analyzes the frequency characteristics of navigation errors of each subsystem and selects the appropriate cut-off frequencies of complementary filters. A semi-physical simulation experiment and result analysis are provided in Section 5. In addition, the content of this paper is summarized in Section 6.

## 2. Coordinate Frames, INS Mechanization and CNS Measurement Transformation

### 2.1. Coordinate Frames in Use

The coordinate frames in use are defined as follows:

1. Navigation coordinate frame (n frame): locally level geographic coordinates, which are defined with its $z_{n}$-axis upward along the local geodetic vertical, $y_{n}$-axis north (and horizon), and $x_{n}$-axis east (and horizon);
2. Body-fixed coordinate frame (b frame): located at the centroid of the vehicle, defined with its $y_{b}$-axis forward along the longitudinal axis of the vehicle, $x_{b}$-axis right, and $z_{b}$-axis upward;
3. Earth-centered Earth-fixed coordinate frame (e frame): located at the center of the Earth, the $x_{e}$-axis extends from the origin to the intersection of the prime meridian (Longitude 0°) and the Equator (Latitude 0°), the $z_{e}$-axis is along the spin axis of the Earth, pointing to the north pole, the $y_{e}$-axis is orthogonal to both the $x_{e}$-axis and $z_{e}$-axis; the three coordinate axes follow the right-handed rule.
4. Earth-centered inertial coordinate frame (i frame): located at the center of the Earth; the $z_{i}$-axis is along the spin axis of the Earth, pointing to the north pole; the $x_{i}$-axis extends from the origin to the spring equinox in the equatorial plane; the $y_{i}$-axis is orthogonal to both the $x_{i}$-axis and $z_{i}$-axis; $x_{i}$, $y_{i}$, and $z_{i}$ follow the right-handed rule.

### 2.2. SINS Mechanization

The navigation parameters of SINS include the attitude quaternion $q_{b}^{n}={\left[\begin{array}{cccc} q_{0} & q_{1} & q_{2} & q_{3} \end{array}\right]}^{T}$, velocity $v_{}^{n}={\left[\begin{array}{ccc} v_{E} & v_{N} & v_{U} \end{array}\right]}^{T}$, and position $p_{}^{n}={\left[\begin{array}{ccc} L & \lambda & h \end{array}\right]}^{T}$; the superscript n denotes the navigation frame and the subscript b denotes the body-fixed frame, $q_{i}$ (i=0,1,2,3) denotes the *i*th component of attitude quaternion. $v_{E}$, $v_{N}$, and $v_{U}$ represent the eastern velocity, northern velocity, and vertical velocity, respectively. L, λ, and h represent the latitude, longitude, and altitude, respectively, and the mechanization equations for SINS are expressed in Equation (1).

(1) $$\left\{ \begin{array}{l} \left[\begin{array}{c} {\dot{q}}_{b}^{n}\\ {\dot{v}}_{}^{n}\\ {\dot{p}}_{}^{n} \end{array}\right]=\left[\begin{array}{c} \frac{1}{2}q_{b}^{n}⊗\omega_{nb}^{b}\\ f_{}^{n}-(\omega_{en}^{n}+2\omega_{ie}^{n})\times v_{}^{n}+g^{n}\\ M_{pv}v_{}^{n} \end{array}\right]\\ M_{pv}=\left[\begin{array}{ccc} 0 & 1/\left(R_{M}+h\right) & 0\\ \sec L/\left(R_{N}+h\right) & 0 & 0\\ 0 & 0 & 1 \end{array}\right] \end{array}\right.$$

where $\omega_{nb}^{b}$ represents the angular velocity of the b frame relative to the n frame expressed in the b frame, $f_{}^{n}$ represents the measurement of a specific force in the n frame, $\omega_{ie}^{n}$ represents the Earth rotation rate denoted in the n frame, $\omega_{en}^{n}$ represents the angular velocity of the n frame relative to the e frame coordinated in the n frame, $g_{}^{n}$ represents the gravity acceleration expressed in the n frame, $R_{M}$ and $R_{N}$ are the radii of curvature in meridian and prime vertical, (L, λ, h) is the position of the vehicle in latitude, longitude and altitude.

### 2.3. CNS Measurement Transformation

The output of the CNS is the attitude quaternion $q_{b}^{i,cns}$ from the b frame to the i frame, but the output of SINS is the attitude quaternion $q_{b}^{n,ins}$ from the b frame to the n frame. If the complementary filter is selected for the information fusion of SINS/CNS integration, the parameter conversion is required firstly; that is to say, $q_{b}^{i,cns}$ (the attitude quaternion from the b frame to the i frame) must be converted into $q_{b}^{n,cns}$ (the attitude quaternion from the b frame to the n frame). The algorithm of CNS quaternion transformation from $q_{b}^{i,cns}$ to $q_{b}^{n,cns}$ is given in this subsection. According to the chain rule, the attitude quaternion $q_{b}^{n,cns}$ is

(2) $$q_{b}^{n,cns}=q_{e}^{n}⊗q_{i}^{e}⊗q_{b}^{i,cns}$$

where $q_{e}^{n}$ is the transformation quaternion from the e frame to the n frame, and $q_{i}^{e}$ is the transformation quaternion from the i frame to the e frame.

#### 2.3.1. The Method for Calculating $q_{e}^{n}$

Given the longitude $\lambda^{gps}$ and latitude $L^{gps}$ of GPS, the position matrix $C_{e}^{n,gps}$ can be calculated according to the relationship between the n frame and e frame, and the expression of $C_{e}^{n,gps}$ is shown in Equation (3):

(3) $$C_{e}^{n,gps}=\left[\begin{array}{ccc} -\sin(\lambda^{gps}) & \cos(\lambda^{gps}) & 0\\ -\sin(L^{gps})cos(\lambda^{gps}) & -\sin(L^{gps})sin(\lambda^{gps}) & \cos(L^{gps})\\ \cos(L^{gps})cos(\lambda^{gps}) & \cos(L^{gps})sin(\lambda^{gps}) & \sin(L^{gps}) \end{array}\right]$$

According to the relationship between the direction cosine matrix and corresponding quaternion, the expression of quaternion $q_{e}^{n}$ is as follows:

(4) $$q_{e}^{n}=Mat2Quat(C_{e}^{n,gps})$$

where Mat2Quat(•) represents the function that calculates the quaternion according to the direction cosine matrix, and its expression is shown in Equation (5).

For any direction cosine matrix $C={\left[T_{ij}^{}\right]}_{3\times 3}$ (i,j=1,2,3), the corresponding attitude quaternion is defined as $q≜{\left[\begin{array}{cccc} q_{0} & q_{1} & q_{2} & q_{3} \end{array}\right]}^{T}$; then, the method of calculating $q_{i}\left(i=0,1,2,3\right)$ is as follows:

(5) $$\left\{ \begin{array}{l} q_{0}=\frac{1}{2}\sqrt{1+T_{11}+T_{22}+T_{33}}\\ q_{1}=\frac{1}{2}\mathrm{sign}\left(T_{32}-T_{23}\right)\sqrt{1+T_{11}-T_{22}-T_{33}}\\ q_{2}=\frac{1}{2}\mathrm{sign}\left(T_{13}-T_{31}\right)\sqrt{1-T_{11}+T_{22}-T_{33}}\\ q_{3}=\frac{1}{2}\mathrm{sign}\left(T_{21}-T_{12}\right)\sqrt{1-T_{11}-T_{22}+T_{33}} \end{array}\right.$$

where sign(•) represents the sign function.

#### 2.3.2. The Method for Calculating $q_{i}^{e}$

The quaternion $q_{i}^{e}$ can be calculated according to the Greenwich Mean Time and Earth rotation rate. Given Greenwich Mean Time $t_{G}$ and Earth rotation rate $\omega_{ie}$, the Greenwich Hour Angle $G_{ST}$ (the rotation angle of the e frame relative to the i frame along the $z_{i}$ axis) is obtained by multiplying $\omega_{ie}$ and $t_{G}$ together:

(6) $$G_{ST}=\omega_{ie}t_{G}$$

Then, the transformation matrix $C_{i}^{e}$ can be calculated, as shown in Equation (7):

(7) $$C_{i}^{e}=\left[\begin{array}{ccc} \cos G_{ST} & \sin G_{ST} & 0\\ -\sin G_{ST} & \cos G_{ST} & 0\\ 0 & 0 & 1 \end{array}\right].$$

According to the relationship between the direction cosine matrix and corresponding attitude quaternion, the quaternion $q_{i}^{e}$ can be calculated using $C_{i}^{e}$, and its expression is shown in Equation (8):

(8) $$q_{i}^{e}=Mat2Quat(C_{i}^{e}).$$

## 3. Design of Complementary Filters for Integrated Navigation

### 3.1. The Principle of Complementary Filter

The complementary filtering method can restrain the error divergence of the integrated navigation system, which is designed according to the frequency-domain characteristics of errors of different subsystems. For any signal variable X, when it is measured using two uncorrelated methods (for convenience, they are denoted as Method #1 and Method #2, respectively), two measurement results $Z_{1}$ and $Z_{2}$ can be obtained, and they can be expressed as:

(9) $$\{\begin{array}{c} Z_{1}=X+V_{1}\\ Z_{2}=X+V_{2} \end{array}$$

where $Z_{1}$ denotes the measurement result using Method #1, $V_{1}$ denotes the measuring error of $Z_{1}$; $Z_{2}$ denotes the measurement result using Method #2, $V_{2}$ denotes the measuring error of $Z_{2}$; $V_{1}$ and $V_{2}$ are assumed to be the low-frequency error and high-frequency error, respectively.

In order to obtain an accurate estimate of variable X, the measurements $Z_{1}$ and $Z_{2}$ are filtered using the high-pass filter (H.P.F. for short) and low-pass filter (L.P.F for short), respectively, so that the errors $V_{1}$ and $V_{2}$ could be eliminated.

In the design of a complementary filter, the transfer functions of H.P.F. and L.P.F are denoted as $G_{1}^{}\left(s\right)$ and $G_{2}^{}\left(s\right)$, respectively. By selecting appropriate parameters for H.P.F. and L.P.F, the condition $G_{1}^{}\left(s\right)+G_{2}^{}\left(s\right)=1$ can be satisfied; then, the estimate $\hat{X}(s)$ of X(s) is obtained by applying the signal reconstruction (S.R.) method (i.e., the sum of the outputs of $G_{1}^{}\left(s\right)$ and $G_{2}^{}\left(s\right)$). For the low-pass filter $G_{2}^{}\left(s\right)$ and high-pass filter $G_{1}^{}\left(s\right)$, the appropriate cut-off frequencies are selected so that $G_{1}^{}\left(s\right)V_{1}\left(s\right)$ and $G_{2}^{}\left(s\right)V_{2}\left(s\right)$ are approximate to zero, and the sum of the outputs of $G_{1}^{}\left(s\right)$ and $G_{2}^{}\left(s\right)$ will be close to the true value of signal variable X, as shown in Equation (10):

(10) $$\hat{X}\left(s\right)=G_{1}^{}\left(s\right)Z_{1}\left(s\right)+G_{2}^{}\left(s\right)Z_{2}\left(s\right)=X\left(s\right)+G_{1}^{}\left(s\right)V_{1}\left(s\right)+G_{2}^{}\left(s\right)V_{2}\left(s\right)\approx X\left(s\right).$$

**Figure 1:**
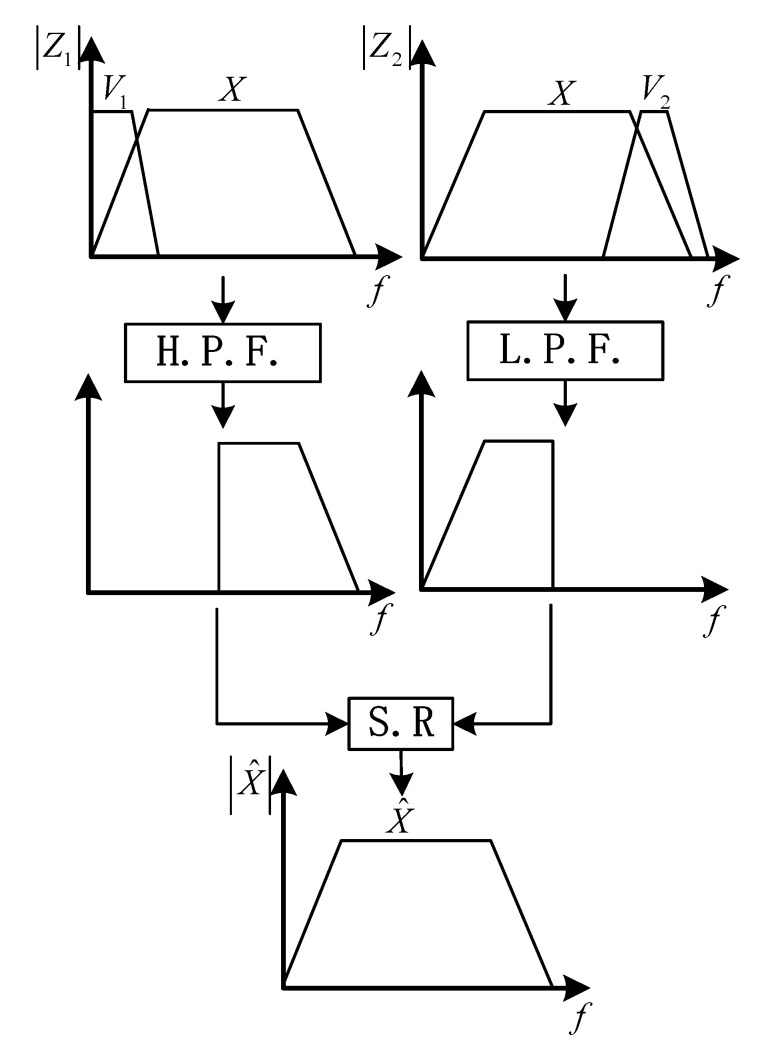
Principle of complementary filter.

The principle of the complementary filter is shown in Figure 1.

### 3.2. Design of Complementary Filters for SINS/CNS/GPS Integration

**Figure 2:**
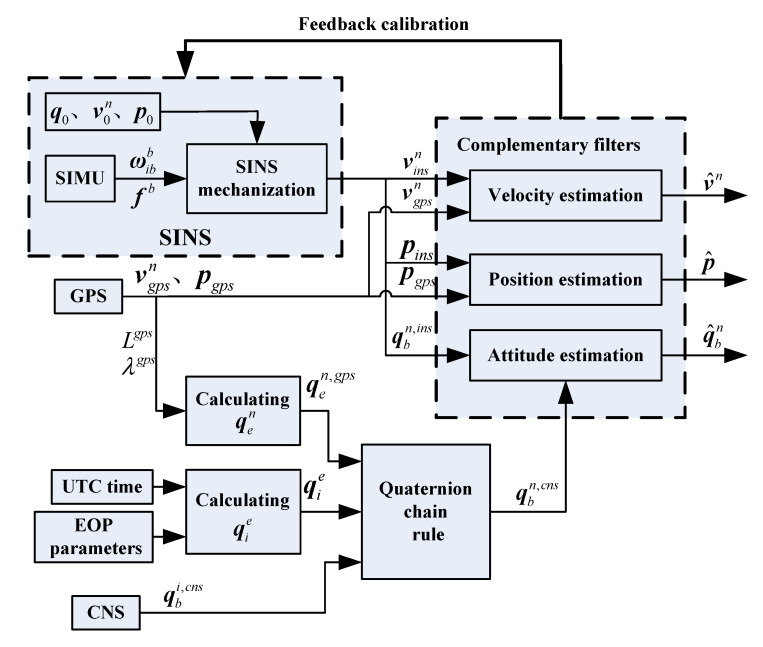
Schematics of strapdown inertial navigation system (SINS)/celestial navigation system (CNS)/global positioning system (GPS) navigation system based on complementary filter.

There are two sources of attitude information for SINS/CNS/GPS integrated navigation: the attitude in the n frame calculated by SINS (denoted as $q_{b}^{n,ins}$), the attitude in the i frame measured by CNS (denoted as $q_{b}^{i,cns}$), and the velocity information sources include the velocity in the n frame calculated by SINS (denoted as $v_{ins}^{n}$) and the velocity in the n frame measured by GPS (denoted as $v_{gps}^{n}\left(s\right)$). Similarly, the position information sources include the position in the n frame calculated by SINS (denoted as $p_{ins}^{}$) and the position in the n frame measured by GPS (denoted as $p_{gps}^{}$). The navigation information (including the attitude, velocity, and position information) obtained by SINS has the advantages of good dynamic performance, high real-time stability, and good short-term stability, but the navigation errors accumulate over time, and the long-term stability is poor. The error sources of SINS mainly include the gyro drifts/accelerometer biases, iterative algorithm errors, initial alignment errors, etc. Due to the cumulative effect of integral calculation, the errors of SINS navigation parameters, which include the attitude error, velocity error, and position error, will accumulate over time, and all the frequency spectrums are mainly in the low frequency segment. The navigation information measured by CNS and GPS (including the attitude from CNS, the velocity and position from GPS) has the advantages of high precision, error convergence, and good long-term stability, but the data update rate is low, and the real-time performance is poor. The measurement errors of CNS and GPS do not accumulate over time and can be approximated as Gaussian white noise. By analyzing the frequency-domain characteristics of the navigation errors of each subsystem, the complementary filters are designed to realize the information fusion of an SINS/CNS/GPS integrated navigation system, and the navigation results with higher precision are obtained. The process of complementary filtering information fusion for an SINS/CNS/GPS integrated navigation system is shown in Figure 2.

#### 3.2.1. Complementary Filter for Attitude Estimation

As shown in Section 2.3, the attitude calculated by SINS is $q_{b}^{n,ins}$, and the attitude measured by CNS is $q_{b}^{i,cns}$. By using the position information of GPS, the attitude of CNS in the n frame can be calculated, as shown in Equation (11):

(11) $$q_{b}^{n,cns}=q_{e}^{n,gps}⊗q_{i}^{e}⊗q_{b}^{i,cns}$$

where $q_{e}^{n,gps}$ represents the transformation quaternion from the e frame to the n frame; $q_{i}^{e}$ represents the transformation quaternion from the i frame to the e frame; $q_{e}^{n,gps}$ can be calculated based on the position of GPS; $q_{i}^{e}$ can be calculated based on the UTC time and EOP parameters, in which the EOP parameters represent the Earth orientation parameters provided by the International Earth Rotation and Reference Systems Service (IERS).

For the attitude estimation, the complementary filter is denoted as CF1, which is composed of a first-order high-pass filter and a first-order low-pass filter. The transfer function of a first-order high-pass filter is denoted as $G_{1}^{H}\left(s\right)=s/\left(s+\omega_{1}^{c}\right)$, and the transfer function of a first-order low-pass filter is denoted as $G_{1}^{L}\left(s\right)=\omega_{1}^{c}/\left(s+\omega_{1}^{c}\right)$, where $\omega_{1}^{c}$ is the cut-off angular frequency of CF1, and the cut-off frequency corresponding to $\omega_{1}^{c}$ is $f_{1}^{c}=\omega_{1}^{c}/\left(2\pi \right)$. Then, the attitude estimation in the s-domain is as follows:

(12) $${\hat{q}}_{b}^{n}\left(s\right)=G_{1}^{H}\left(s\right)q_{b}^{n,ins}\left(s\right)+G_{1}^{L}\left(s\right)q_{b}^{n,cns}\left(s\right).$$

Suppose that the filtering period is $T_{f}^{}=t_{k}^{}-t_{k-1}^{}$. Discretize Equation (12) to obtain the recursive form in time domain for attitude estimation, as shown in Equation (13):

(13) $${\hat{q}}_{b}^{n}\left(t_{k}^{}\right)=\frac{1}{1+\omega_{1}^{c}T_{f}}{\hat{q}}_{b}^{n}\left(t_{k-1}^{}\right)+\frac{T_{f}}{1+\omega_{1}^{c}T_{f}}{\dot{q}}_{b}^{n,ins}\left(t_{k}^{}\right)+\frac{\omega_{1}^{c}T_{f}}{1+\omega_{1}^{c}T_{f}}q_{b}^{n,cns}\left(t_{k}^{}\right)$$

where $\omega_{1}^{c}$ represents the angular frequency corresponding to CF1′s cut-off frequency $f_{1}^{c}=\omega_{1}^{c}/\left(2\pi \right)$.

#### 3.2.2. Complementary Filter for Velocity Estimation

As mentioned, the velocity of SINS is $v_{ins}^{n}$, and the velocity of GPS is $v_{gps}^{n}$. For the velocity estimation, the complementary filter is denoted as CF2, which is composed of a first-order high-pass filter and a first-order low-pass filter. The transfer function of a first-order high-pass filter is denoted as $G_{2}^{H}\left(s\right)=s/\left(s+\omega_{2}^{c}\right)$, and the transfer function of a first-order low-pass filter is denoted as $G_{2}^{L}\left(s\right)=\omega_{2}^{c}/\left(s+\omega_{2}^{c}\right)$, where $\omega_{2}^{c}$ represents the angular frequency corresponding to CF2′s cut-off frequency $f_{2}^{c}=\omega_{2}^{c}/\left(2\pi \right)$. Then, the velocity estimation in the s-domain is as follows:

(14) $${\hat{v}}_{}^{n}\left(s\right)=G_{2}^{H}\left(s\right)v_{ins}^{n}\left(s\right)+G_{2}^{L}\left(s\right)v_{gps}^{n}\left(s\right).$$

Similarly, the filtering period is also $T_{f}$. Discretize Equation (14) to obtain the recursive form in the time domain for velocity estimation, as shown in Equation (15):

(15) $${\hat{v}}_{}^{n}\left(t_{k}^{}\right)=\frac{1}{1+\omega_{2}^{c}T_{f}}{\hat{v}}_{}^{n}\left(t_{k-1}^{}\right)+\frac{T_{f}}{1+\omega_{2}^{c}T_{f}}{\dot{v}}_{ins}^{n}\left(t_{k}^{}\right)+\frac{\omega_{2}^{c}T_{f}}{1+\omega_{2}^{c}T_{f}}v_{gps}^{n}\left(t_{k}^{}\right).$$

#### 3.2.3. Complementary Filter for Position Estimation

For position estimation, the position of SINS is $p_{ins}$, the position of GPS is $p_{gps}$, and the complementary filter is denoted as CF3, which is also composed of a first-order high-pass filter and a first-order low-pass filter. The transfer function of a first-order high-pass filter is $G_{3}^{H}\left(s\right)=s/\left(s+\omega_{3}^{c}\right)$, and the transfer function of a first-order low-pass filter is $G_{3}^{L}\left(s\right)=\omega_{3}^{c}/\left(s+\omega_{3}^{c}\right)$, where $\omega_{3}^{c}$ is the angular frequency corresponding to CF3′s cut-off frequency $f_{3}^{c}=\omega_{3}^{c}/\left(2\pi \right)$. Then, the position estimation in the s-domain is as follows:

(16) $$\hat{p}\left(s\right)=G_{3}^{H}\left(s\right)p_{ins}^{}\left(s\right)+G_{3}^{L}\left(s\right)p_{gps}^{}\left(s\right).$$

Similarly, take the filtering period $T_{f}^{}$ as a discrete step, and discretize Equation (16) to obtain the recursive form in the time domain for position estimation, as shown in Equation (17):

(17) $$\hat{p}\left(t_{k}^{}\right)=\frac{1}{1+\omega_{3}^{c}T_{f}}\hat{p}\left(t_{k-1}^{}\right)+\frac{T_{f}}{1+\omega_{3}^{c}T_{f}}{\dot{p}}_{ins}^{}\left(t_{k}^{}\right)+\frac{\omega_{3}^{c}T_{f}}{1+\omega_{3}^{c}T_{f}}p_{gps}^{}\left(t_{k}^{}\right).$$

Each time after the process of complementary filtering is completed, the navigation parameters of SINS are corrected by feedback of the estimation results, as shown in Equation (18):

(18) $$\left\{ \begin{array}{l} q_{b}^{n,ins}\left(t_{k}^{}\right)={\hat{q}}_{b}^{n}\left(t_{k}^{}\right)\\ v_{ins}^{n}\left(t_{k}^{}\right)={\hat{v}}_{}^{n}\left(t_{k}^{}\right)\\ p_{ins}^{}\left(t_{k}^{}\right)=\hat{p}\left(t_{k}^{}\right) \end{array}\right..$$

## 4. Error Analysis in Frequency Domain and Selection of Cut-Off Frequency 

The cut-off frequency is the most key parameter of a complementary filter, which determines the effect of information fusion. In order to obtain good estimation results, it is necessary to select the cut-off frequencies properly for the complementary filters by analyzing the errors of each navigation subsystem in the frequency domain. According to the principle of complementary filter in Section 3.1, the analysis shows that the selection of cut-off frequency of the complementary filter determines the influential weight of each navigation subsystem (SINS, CNS, or GPS) on the SINS/CNS /GPS integrated navigation system. In conclusion, the larger the cut-off frequency $f_{i}^{c}$ (i=1,2,3), the greater the influence of the measurement noise of the auxiliary subsystem (CNS or GPS) on the estimation result, the smaller the influence of the errors of SINS, and vice versa. During the on-orbit flight of an aerospace plane, the navigation errors of CNS and GPS are mainly the measurement noises, which can be approximated as Gaussian white noise. Under the premise of satisfying the dynamic performance requirements of SINS, the cut-off frequency of the complementary filter should be as small as possible, so that the measurement noise errors of CNS and GPS can be filtered out in the largest frequency range. In this paper, the errors of each subsystem are analyzed in the time and frequency domain. According to the relation between the cut-off frequencies and statistical errors, the cut-off frequencies of complementary filters for attitude/velocity/position estimation are optimally selected, respectively.

### 4.1. Selection of Cut-Off Frequency Based on Nonlinear Optimization Theory

In this subsection, the analysis method based on power spectral density is used to analyze the influence of the noise of each subsystem on the output noise of the SINS/CNS/GPS integrated navigation system theoretically, and an optimal selection scheme of the cut-off frequency of the complementary filter based on the constrained nonlinear optimization method is proposed, so as to provide a theoretical reference for the selection of the optimal cut-off frequency.

In SINS/CNS/GPS integrated navigation, the measurement noises of navigation sensors such as gyro, accelerometer, CNS, and GPS are all stationary random noise. For the convenience of theoretical analysis, these noises are assumed to be Gaussian white noise with zero mean. In this paper, the measurement noises of a gyro, accelerometer, CNS, GPS velocity, and GPS position are denoted as $w_{gro}\left(t\right)$, $w_{acc}\left(t\right)$, $w_{cns\_att}\left(t\right)$, $w_{gps\_vel}\left(t\right)$, and $w_{gps\_pos}\left(t\right)$, respectively. The random walk noises in SINS attitude, velocity, and position are denoted as $w_{ins\_att}\left(t\right)$, $w_{ins\_vel}\left(t\right)$, and $w_{ins\_pos}\left(t\right)$. $q_{w_{gro}}$, $q_{w_{acc}}$, $q_{w_{cns\_att}}$, $q_{w_{gps\_vel}}$, and $q_{w_{gps\_pos}}$ represent the variance intensities of gyro angular velocity measurement noise, accelerometer specific force measurement noise, CNS attitude measurement noise, GPS velocity measurement noise, and GPS position measurement noise, respectively. δ() represents the Dirac delta function. Then, the covariances and autocorrelation functions of measurement noises of navigation sensors are expressed as:

(19) $$\{\begin{array}{ll} E\left[w_{gro}\left(t\right)w_{gro}^{T}\left(\tau \right)\right]=q_{w_{gro}}\delta \left(t-\tau \right) & R_{w_{gro}}\left(\mu \right)=q_{w_{gro}}\delta \left(\mu \right)\\ E\left[w_{acc}\left(t\right)w_{acc}^{T}\left(\tau \right)\right]=q_{w_{acc}}\delta \left(t-\tau \right) & R_{w_{acc}}\left(\mu \right)=q_{w_{acc}}\delta \left(\mu \right)\\ E\left[w_{cns\_att}\left(t\right)w_{cns\_att}^{T}\left(\tau \right)\right]=q_{cns\_att}\delta \left(t-\tau \right) & R_{w_{cns\_att}}\left(\mu \right)=q_{w_{cns\_att}}\delta \left(\mu \right)\\ E\left[w_{gps\_vel}\left(t\right)w_{gps\_vel}^{T}\left(\tau \right)\right]=q_{gps\_vel}\delta \left(t-\tau \right) & R_{w_{gps\_vel}}\left(\mu \right)=q_{w_{gps\_vel}}\delta \left(\mu \right)\\ E\left[w_{gps\_pos}\left(t\right)w_{gps\_pos}^{T}\left(\tau \right)\right]=q_{gps\_pos}\delta \left(t-\tau \right) & R_{w_{gps\_pos}}\left(\mu \right)=q_{w_{gps\_pos}}\delta \left(\mu \right) \end{array}$$

where μ=t−τ represents the time interval between the time instants t and τ.

The power spectral density functions of measurement noises of these navigation sensors are

(20) $$\{\begin{array}{l} S_{w_{gro}}\left(\omega \right)=F\left\{R_{w_{gro}}\left(\mu \right)\right\}=\int_{-\infty }^{\infty }q_{w_{gro}}\delta \left(\mu \right)e^{-j\omega \mu }d\mu =q_{w_{gro}}\\ S_{w_{acc}}\left(\omega \right)=F\left\{R_{w_{acc}}\left(\mu \right)\right\}=\int_{-\infty }^{\infty }q_{w_{acc}}\delta \left(\mu \right)e^{-j\omega \mu }d\mu =q_{w_{acc}}\\ S_{w_{cns\_att}}\left(\omega \right)=F\left\{R_{w_{cns\_att}}\left(\mu \right)\right\}=\int_{-\infty }^{\infty }q_{w_{cns\_att}}\delta \left(\mu \right)e^{-j\omega \mu }d\mu =q_{w_{cns\_att}}\\ S_{w_{gps\_vel}}\left(\omega \right)=F\left\{R_{w_{gps\_vel}}\left(\mu \right)\right\}=\int_{-\infty }^{\infty }q_{w_{gps\_vel}}\delta \left(\mu \right)e^{-j\omega \mu }d\mu =q_{w_{gps\_vel}}\\ S_{w_{gps\_pos}}\left(\omega \right)=F\left\{R_{w_{gps\_pos}}\left(\mu \right)\right\}=\int_{-\infty }^{\infty }q_{w_{gps\_pos}}\delta \left(\mu \right)e^{-j\omega \mu }d\mu =q_{w_{gps\_pos}} \end{array}$$

where F{} is the Fourier transform.

For the convenience of analysis, the motion state of the vehicle and the random error propagation model of SINS are simplified. Assuming that the vehicle is in an on-orbit cruise phase and its motion state is uniform straight-line flight, then the error model of SINS can be simplified as a linear time-invariant system.

The attitude random walk noise $w_{ins\_att}\left(t\right)$ of SINS is the integral of gyro measurement noise $w_{gro}\left(t\right)$, and the frequency response function of the integrator is 1/(jω). According to the response characteristics of the linear system to stationary process, the power spectral density of $w_{ins\_att}$ is

(21) $$S_{w_{ins\_att}}\left(\omega \right)={\left|1/\left(j\omega \right)\right|}^{2}S_{w_{gro}}\left(\omega \right)=q_{w_{gro}}/\omega^{2}.$$

Similarly, the SINS velocity random walk noise $w_{ins\_vel}$ is the integral of measurement noise of the accelerometer, and the power spectral density of $w_{ins\_vel}$ is

(22) $$S_{w_{ins\_vel}}\left(\omega \right)={\left|1/\left(j\omega \right)\right|}^{2}S_{w_{acc}}\left(\omega \right)=q_{w_{acc}}/\omega^{2}.$$

Due to the measurement noises of inertial sensors and the calculation error of the SINS update algorithm, there will be white noise in the velocity information of SINS, which could be denoted as $w_{ins\_nv}^{}$, and its variance intensity is denoted as $q_{w_{ins\_nv}}$. The power spectral density of $w_{ins\_nv}^{}$ is $S_{w_{ins\_nv}}\left(\omega \right)=q_{w_{ins\_nv}}$, and the position random walk noise $w_{ins\_pos}$ of SINS is the integral of velocity white noise; its power spectral density is as follows:

(23) $$S_{w_{ins\_pos}}\left(\omega \right)={\left|1/\left(j\omega \right)\right|}^{2}S_{w_{ins\_nv}}\left(\omega \right)=q_{w_{ins\_nv}}/\omega_{}^{2}.$$

In this paper, the random errors of CF1, CF2, and CF3 are denoted as $w_{CF\_att}$, $w_{CF\_vel}$, and $w_{CF\_pos}$, respectively. Firstly, the power spectral density of a random error of attitude estimation complementary filter CF1 is analyzed. According to the design of complementary filters in Section 3.2, the transfer functions of the high-pass filter and low-pass filter of CF1 are rewritten as follows:

(24) $$\left\{ \begin{array}{l} G_{1}^{H}\left(s\right)=s/\left(s+\omega_{1}^{c}\right)\\ G_{1}^{L}\left(s\right)=\omega_{1}^{c}/\left(s+\omega_{1}^{c}\right) \end{array}\right..$$

From the transfer functions, the corresponding frequency response functions can be obtained as follows:

(25) $$\left\{ \begin{array}{l} G_{1}^{H}\left(j\omega \right)={G_{1}^{H}\left(s\right)|}_{s=j\omega }=j\omega /\left(j\omega +\omega_{1}^{c}\right)\\ G_{1}^{L}\left(j\omega \right)={G_{1}^{L}\left(s\right)|}_{s=j\omega }=\omega_{1}^{c}/\left(j\omega +\omega_{1}^{c}\right) \end{array}\right..$$

According to the principle of complementary filter, the random error of CF1 is the sum of two random errors, of which one is the output of $G_{1}^{H}\left(j\omega \right)$ under the input of SINS attitude random walk noise, and the other is the output of $G_{1}^{L}\left(j\omega \right)$ under the input of CNS attitude measurement noise. Since SINS and CNS are two independent subsystems, the random error of SINS is orthogonal to that of CNS, so the power spectral density $S_{w_{CF\_att}}\left(\omega \right)$ of the random error of CF1 is

(26) $$S_{w_{CF\_att}}\left(\omega \right)={\left|G_{1}^{H}\left(j\omega \right)\right|}_{}^{2}S_{w_{ins\_att}}\left(\omega \right)+{\left|G_{1}^{L}\left(j\omega \right)\right|}_{}^{2}S_{w_{cns\_att}}\left(\omega \right).$$

Substituting $G_{1}^{H}\left(j\omega \right)$, $G_{1}^{L}\left(j\omega \right)$, $S_{w_{ins\_att}}\left(\omega \right)$, and $S_{w_{cns\_att}}\left(\omega \right)$ into Equation (26), we can obtain

(27) $$S_{w_{CF\_att}}\left(\omega \right)={\left|j\omega /\left(j\omega +\omega_{1}^{c}\right)\right|}_{}^{2}q_{w_{gro}}/\omega^{2}+{\left|\omega_{1}^{c}/j\omega +\omega_{1}^{c}\right|}_{}^{2}q_{w_{cns\_att}}=\left(q_{w_{gro}}+q_{w_{cns\_att}}{\left(\omega_{1}^{c}\right)}_{}^{2}\right)/\left(\omega_{}^{2}+{\left(\omega_{1}^{c}\right)}_{}^{2}\right).$$

By substituting ω=2πf into Equation (27), the expression of the power spectral density function of $w_{CF\_att}$ with respect to frequency f can be obtained.

(28) $$S_{w_{CF\_att}}\left(f\right)={S_{w_{CF\_att}}\left(\omega \right)|}_{\omega =2\pi f}^{}=\left(q_{w_{gro}}+q_{w_{cns\_att}}{\left(\omega_{1}^{c}\right)}_{}^{2}\right)/\left({\left(2\pi f\right)}_{}^{2}+{\left(\omega_{1}^{c}\right)}_{}^{2}\right)$$

In a SINS/CNS/GPS integrated navigation system, the maximum frequency of a filter’s bandwidth is limited due to the discrete sampling of a signal. Among the three subsystems of SINS, CNS, and GPS, the sampling frequency $f_{ins}^{s}$ of SINS is the highest. According to the sampling theorem of a stationary random process, the power spectral density of the output noise of a SINS/CNS/GPS integrated navigation system only needs to be considered in the frequency range of $\left[0,f_{ins}^{s}/2\right]$. By calculating the definite integral of $S_{w_{CF\_att}}\left(f\right)$ with respect to f from 0 to $f_{s}^{ins}/2$, the average power $P_{w_{CF\_att}}$ of the random error $w_{CF\_att}$ of CF1 in the frequency band of $\left[0,f_{s}^{ins}/2\right]$ could be obtained, as shown in Equation (29):

(29) $$P_{w_{CF\_att}}=\int_{0}^{f_{s}^{ins}/2}S_{w_{CF\_att}}\left(f\right)df=\arctan\left(\pi f_{s}^{ins}/\omega_{1}^{c}\right)\left(q_{w_{gro}}+q_{w_{cns\_att}}{\left(\omega_{1}^{c}\right)}_{}^{2}\right)/\left(2\pi \omega_{1}^{c}\right).$$

By substituting $\omega_{1}^{c}=2\pi f_{1}^{c}$ into Equation (29), the expression of average power $P_{w_{CF\_att}}$ with respect to the cut-off frequency $f_{1}^{c}$ can be obtained, as shown in Equation (30):

(30) $$P_{w_{CF\_att}}=\arctan\left(f_{s}^{ins}/\left(2f_{1}^{c}\right)\right)\left(4q_{w_{cns\_att}}\pi_{}^{2}{\left(f_{1}^{c}\right)}_{}^{2}+q_{w_{gro}}\right)/\left(4\pi_{}^{2}f_{1}^{c}\right).$$

In the algorithm design of a practical integrated navigation system, the parameters of $f_{s}^{ins}$, $q_{w_{gro}}$, and $q_{w_{cns\_att}}$ are constant, and $P_{w_{CF\_att}}$ is a nonlinear function of $f_{1}^{c}$. The optimal value of $f_{1}^{c}$ is equivalent to the value that minimizes $P_{w_{CF\_att}}$. By differentiating $P_{w_{CF\_att}}$ with respect to $f_{1}^{c}$, we can obtain

(31) $$\begin{array}{ccc} \frac{dP_{w_{CF\_att}}}{df_{1}^{c}} & = & 2q_{w_{cns\_att}}\arctan\left(f_{s}^{ins}/\left(2f_{1}^{c}\right)\right)\\ & & -\left(\arctan\left(f_{s}^{ins}/\left(2f_{1}^{c}\right)\right)\left(4q_{w_{cns\_att}}\pi_{}^{2}{\left(f_{1}^{c}\right)}_{}^{2}+q_{w_{gro}}\right)\right)/\left(4\pi_{}^{2}{\left(f_{1}^{c}\right)}_{}^{2}\right)\\ & & -\left(f_{s}^{ins}\left(4q_{w_{cns\_att}}\pi_{}^{2}{\left(f_{1}^{c}\right)}_{}^{2}+q_{w_{gro}}\right)\right)/\left(8\pi_{}^{2}{\left(f_{1}^{c}\right)}_{}^{3}\left({\left(f_{s}^{ins}\right)}^{2}/\left(4{\left(f_{1}^{c}\right)}_{}^{2}\right)+1\right)\right). \end{array}$$

It can be seen from Equation (31) that the derivative of $P_{w_{CF\_att}}$ with respect to $f_{1}^{c}$ has a very complex form, so it is difficult to find the minimum value of $P_{w_{CF\_att}}$ in analytical form by using the stationary point method. Considering that in a specific SINS/CNS/GPS integrated navigation system, the values of $f_{s}^{ins}$, $q_{w_{gro}}$, $q_{w_{cns\_att}}$ are known or can be obtained by calibration, thus, the minimum value of $P_{w_{CF\_att}}$ with respect to $f_{1}^{c}$ can be solved by the optimization method, and the nonlinear optimization function of the MATLAB Optimization Toolbox can be used to obtain the numerical solution of the optimization problem.

Combined with optimization theory, the optimal solution of $f_{1}^{c}$ can be regarded as a constrained nonlinear optimization problem, and the optimal mathematical model is established as

(32) $$\left\{ \begin{array}{l} \underset{f_{1}^{c}}{\min}P_{w_{CF\_att}}=\arctan\left(f_{s}^{ins}/\left(2f_{1}^{c}\right)\right)\left(4q_{w_{cns\_att}}\pi_{}^{2}{\left(f_{1}^{c}\right)}_{}^{2}+q_{w_{gro}}\right)/\left(4\pi_{}^{2}f_{1}^{c}\right)\\ s.t.f_{L}^{c}\leq f_{1}^{c}\leq f_{s}^{cns}/2 \end{array}\right.$$

where $f_{L}^{c}$ is the infimum of the range of the CF’s cut-off frequency, which can be determined by analyzing the error propagation characteristics of the SINS. Under the condition of $f_{1}^{c}\geq f_{L}^{c}$, the high-pass filter of CF1 can effectively restrain the oscillation and divergence low-frequency errors in the SINS attitude. Many studies about inertial navigation analyzed the error propagation characteristics of the inertial navigation system, which are not outlined here. Furthermore, the selection of $f_{1}^{c}$ should meet the requirement of “minimizing the influence of measurement noise of CNS”, thus $f_{1}^{c}\leq f_{s}^{cns}/2$, i.e., the constraint condition in Equation (32). For programming implementation, the MATLAB built-in function “fmincon” can be used to solve Equation (32).

The power spectral density analyses of random errors of CF2 and CF3 are similar to that of CF1. The detailed analysis process will not be introduced here, the analysis results of random noises of CF2 and CF3 are given directly (including the power spectral density, average power, and optimal mathematical model).

According to the power spectral density analysis of random noise of CF2, the following conclusions can be obtained:

Power spectral density of random error of CF2 $S_{w_{CF\_vel}}\left(f\right)$:

(33) $$S_{w_{CF\_vel}}\left(f\right)=\left(q_{w_{acc}}+q_{w_{gps\_vel}}{\left(\omega_{2}^{c}\right)}_{}^{2}\right)/\left({\left(2\pi f\right)}_{}^{2}+{\left(\omega_{2}^{c}\right)}_{}^{2}\right).$$

Average power of random error of CF2 $P_{w_{CF\_vel}}$:

(34) $$P_{w_{CF\_vel}}=\arctan\left(f_{s}^{ins}/\left(2f_{2}^{c}\right)\right)\left(4q_{w_{gps\_vel}}\pi_{}^{2}{\left(f_{2}^{c}\right)}_{}^{2}+q_{w_{acc}}\right)/\left(4\pi_{}^{2}f_{2}^{c}\right).$$

In order to solve the optimization problem of $f_{2}^{c}$, the mathematical model of constrained nonlinear optimization is established as follows:

(35) $$\left\{ \begin{array}{l} \underset{f_{2}^{c}}{\min}P_{w_{CF\_vel}}=\arctan\left(f_{s}^{ins}/\left(2f_{2}^{c}\right)\right)\left(4q_{w_{gps\_vel}}\pi_{}^{2}{\left(f_{2}^{c}\right)}_{}^{2}+q_{w_{acc}}\right)/\left(4\pi_{}^{2}f_{2}^{c}\right)\\ s.t.f_{L}^{c}\leq f_{2}^{c}\leq f_{s}^{gps}/2 \end{array}\right.$$

where $f_{L}^{c}$ is the spectrum bandwidth of INS errors. Under the condition of $f_{2}^{c}\geq f_{L}^{c}$, the high-pass filter of CF2 can effectively eliminate the oscillation and divergence terms of SINS velocity errors. $f_{s}^{gps}$ is the sampling frequency of GPS.

According to the power spectral density analysis of random noise of CF3, the following conclusions can be obtained:

Power spectral density of random error of CF3 $S_{w_{CF\_pos}}\left(f\right)$:

(36) $$S_{w_{CF\_pos}}\left(f\right)=\left(q_{w_{ins\_nv}}+q_{w_{gps\_pos}}{\left(\omega_{3}^{c}\right)}_{}^{2}\right)/\left({\left(2\pi f\right)}_{}^{2}+{\left(\omega_{3}^{c}\right)}_{}^{2}\right).$$

Average power of random error of CF3 $P_{w_{CF\_pos}}$:

(37) $$P_{w_{CF\_pos}}=\arctan\left(f_{s}^{ins}/\left(2f_{3}^{c}\right)\right)\left(q_{w_{gps\_pos}}{\left(2\pi f_{3}^{c}\right)}_{}^{2}+q_{w_{ins\_nv}}\right)/\left(4\pi_{}^{2}f_{3}^{c}\right).$$

To solve the optimization problem of $f_{3}^{c}$, the mathematical model of constrained nonlinear optimization is established as follows:

(38) $$\left\{ \begin{array}{l} \underset{f_{3}^{c}}{\min}P_{w_{CF\_pos}}=\arctan\left(f_{s}^{ins}/\left(2f_{3}^{c}\right)\right)\left(q_{w_{gps\_pos}}{\left(2\pi f_{3}^{c}\right)}_{}^{2}+q_{w_{ins\_nv}}\right)/\left(4\pi_{}^{2}f_{3}^{c}\right)\\ s.t.f_{L}^{c}\leq f_{3}^{c}\leq f_{s}^{gps}/2 \end{array}\right.$$

where $f_{L}^{c}$ is the spectrum bandwidth of SINS errors. Under the condition of $f_{3}^{c}\geq f_{L}^{c}$, the high-pass filter of CF3 can effectively eliminate the oscillation and divergence terms in INS position errors. $f_{s}^{gps}$ is the sampling frequency of GPS.

### 4.2. Selection of Cut-Off Frequency Based on Simulation Test

#### 4.2.1. Spectrum Analysis Based on DFT 

According to the theory of digital signal processing, the discrete Fourier transform (DFT) is often used for signal spectrum analysis. If an N-point time-domain sampling sequence is denoted as x(n) (0≤n≤N−1), then the corresponding N-point DFT of x(n) is defined as:

(39) $$X\left(k\right)=\mathrm{DFT}\left[x\left(n\right)\right]=\sum_{n=0}^{N-1}x\left(n\right)W_{N}^{nk}0\leq k\leq N$$

where $W_{N}=\exp\left(-j2\pi /N\right)$ is the twiddle factor.

In order to study the frequency distribution of error, DFT is often used to analyze the frequency spectrum of an error signal, mainly focusing on the amplitude-frequency characteristics. For DFT, the sampling frequency of the signal is denoted as $f_{s}$; then, the steps of Fourier analysis on the N-point sampling data x(n) are as follows:

Step 1: The DFT transform on the N-point sampling data x(n) is performed to obtain the N-point DFT X(k).

Step 2: Calculate the spectral amplitude |X(k)| for the spectrum data X(k) of the ordinal number 0~fix(N/2), where fix(•) denotes the function rounding a number to the nearest integer.

Step 3: Divide all amplitude data of the spectra obtained in Step 2 by N; then, multiply the amplitude data of ordinal number 2~fix(N/2)+1 by 2, convert the two-sided spectrum into a one-sided spectrum, and plot the amplitude-frequency curve.

Step 4: The frequencies of spectral lines labeled on the abscissa axis of the amplitude-frequency figure are sequentially $kf_{s}/N$ Hz, (k=0~fix(N/2)).

Step 5: The ordinate of spectral line corresponds to the harmonic amplitude of sampling signal x(n) at the corresponding frequency, the unit of amplitude is the same as that of time-domain sampling data, and the amplitude of DC component is at zero frequency.

#### 4.2.2. Selection of Cut-Off Frequencies Based on Simulation Test

**Figure 3:**
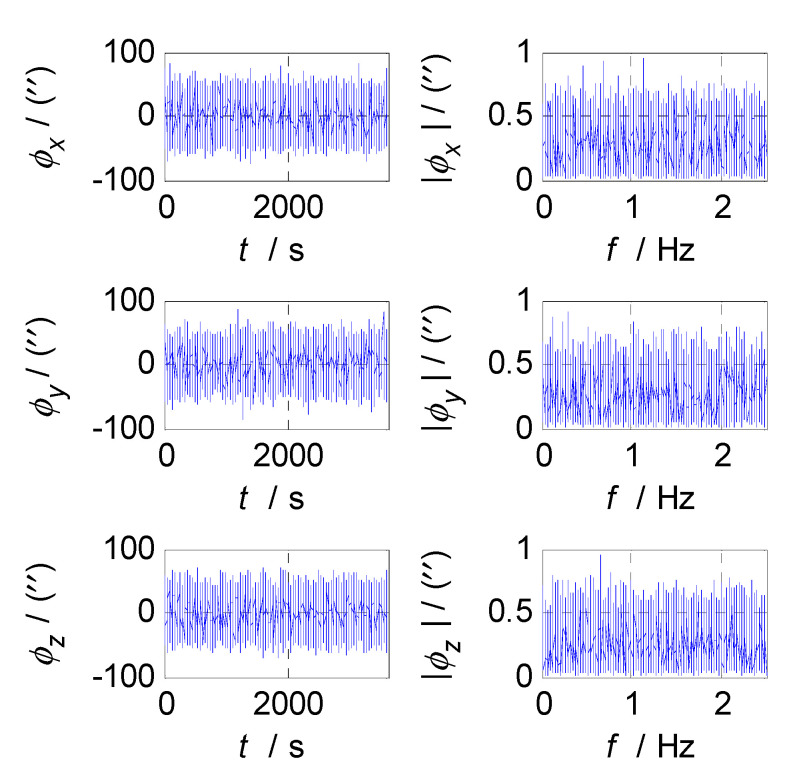
Attitude errors and frequency characteristics of CNS.

**Figure 4:**
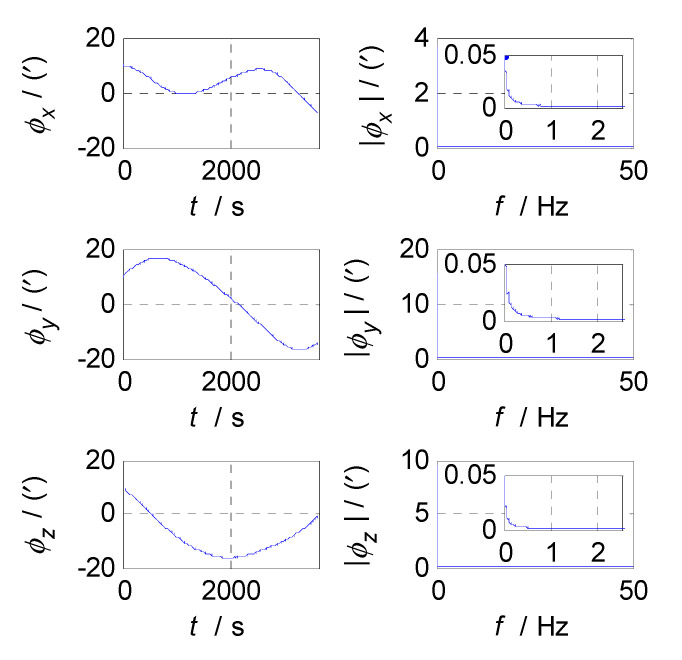
Attitude errors and frequency characteristics of SINS.

**Figure 5:**
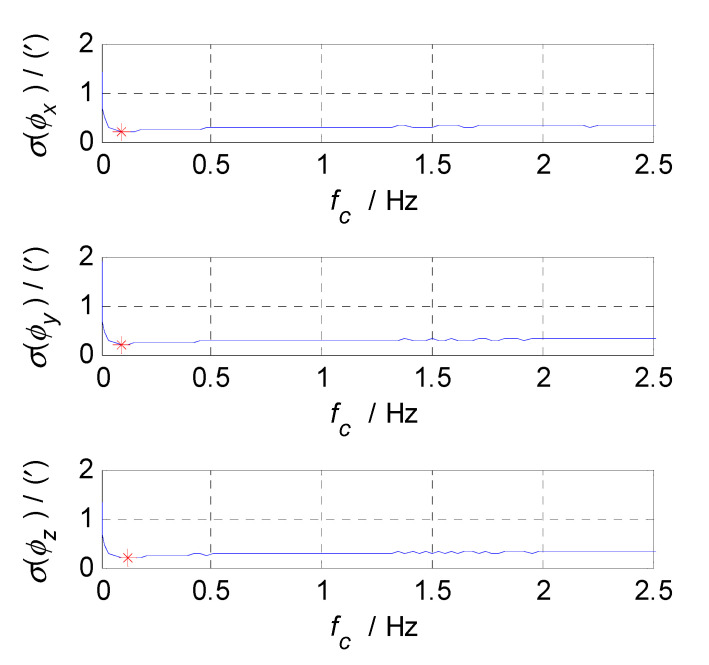
The relationship between $f_{1}^{c}$ and $\sigma \left(\phi_{i}\right)$.

**Figure 6:**
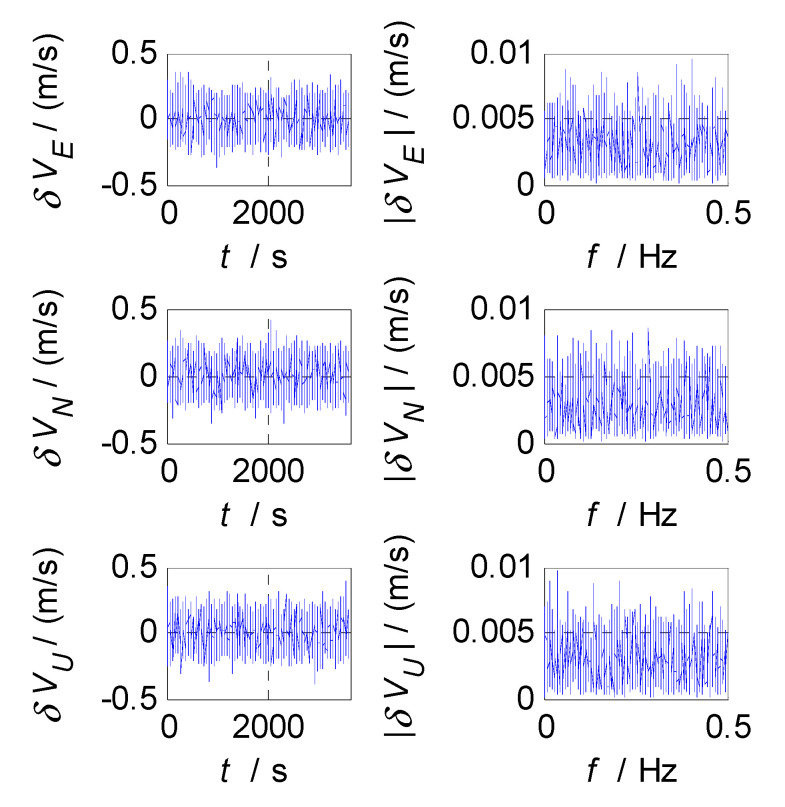
Velocity errors and frequency characteristics of GPS.

**Figure 7:**
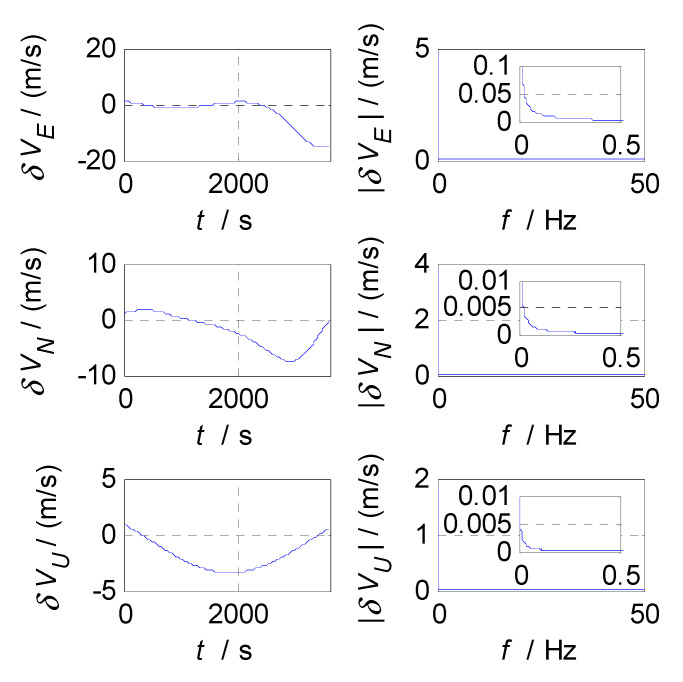
Velocity errors and frequency characteristics of SINS.

**Figure 8:**
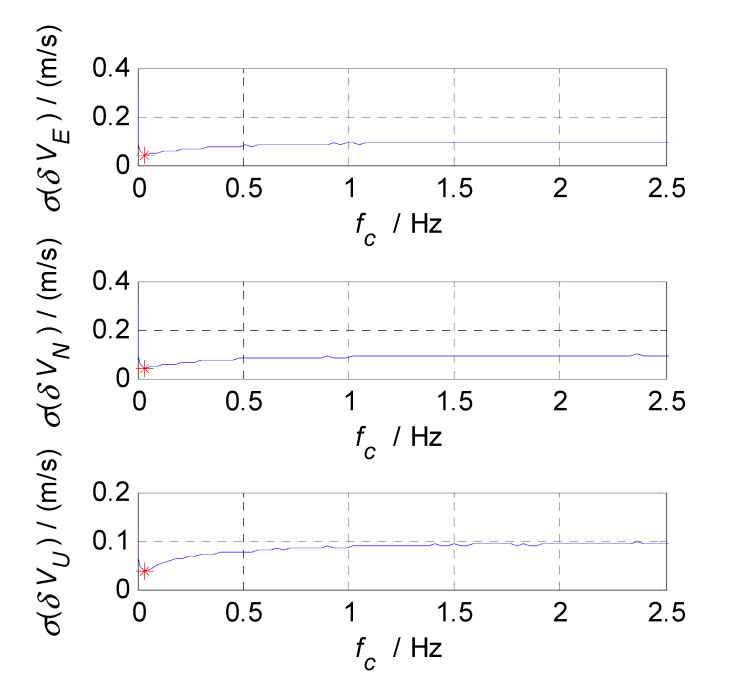
The relationship between $f_{2}^{c}$ and $\sigma \left(\delta V_{i}\right)$.

**Figure 9:**
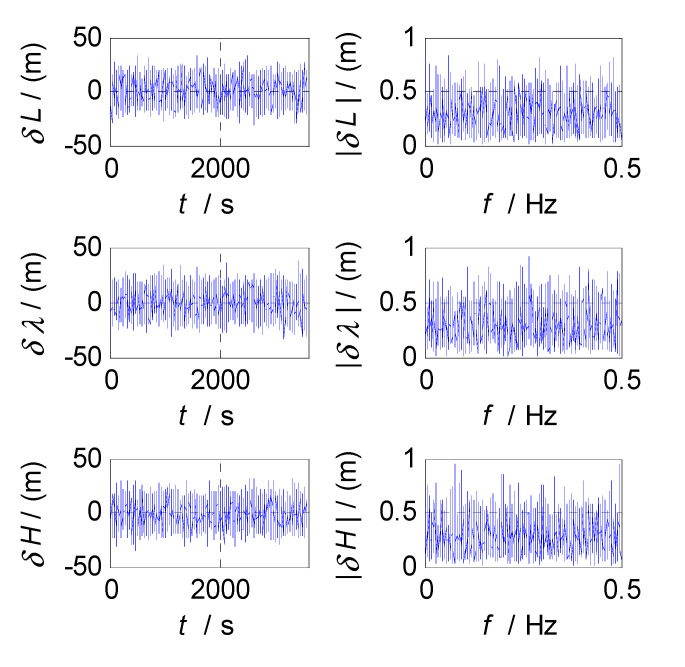
Position errors and frequency characteristics of GPS.

**Figure 10:**
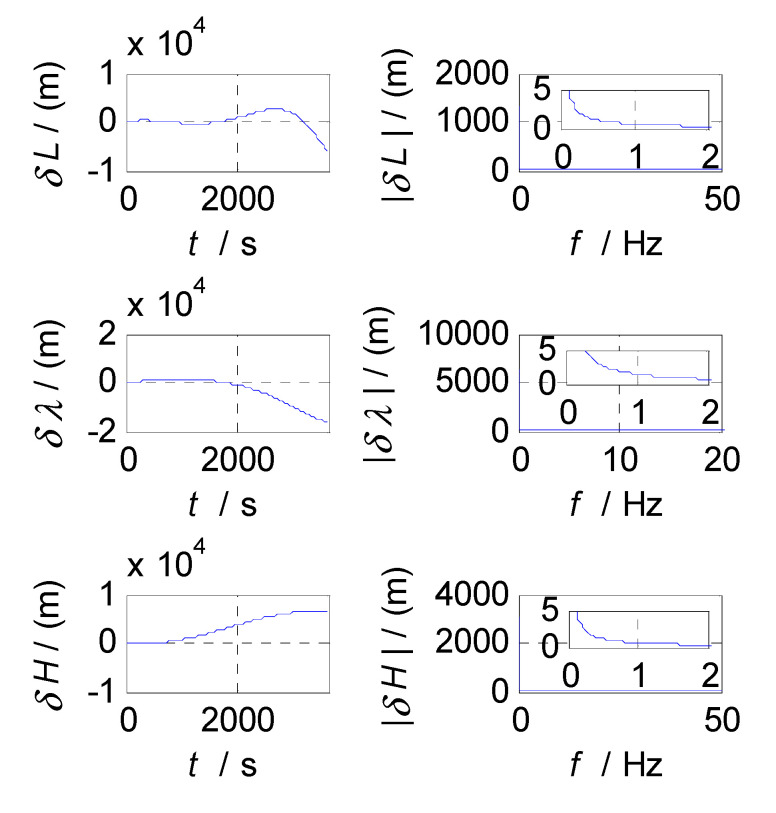
Position errors and frequency characteristics of SINS.

**Figure 11:**
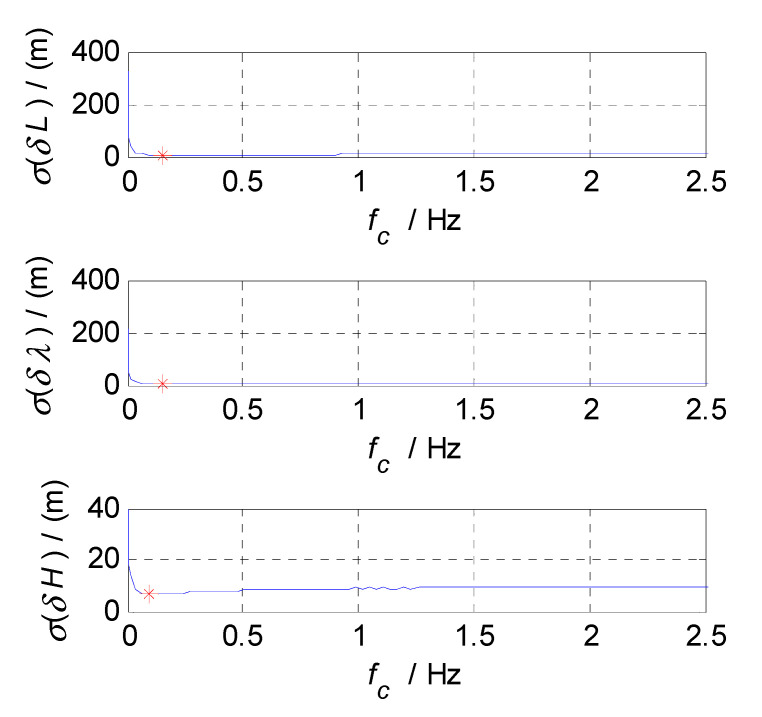
The relationships between $f_{3}^{c}$ and $\sigma \left(\delta P_{i}\right)$.

In order to determine the cut-off frequencies of complementary filters for the attitude/velocity/position estimation in an SINS/CNS/GPS integrated navigation system, the frequency-domain analyses were conducted on the measurement errors of SINS, CNS, and GPS subsystems. The analysis results, which include the error curves, amplitude-frequency diagrams, and relationship graphs between the estimation errors and selected cut-off frequencies, are shown in Figure 3, Figure 4, Figure 5, Figure 6, Figure 7, Figure 8, Figure 9, Figure 10 and Figure 11, respectively.

Firstly, analyze and select the cut-off frequency of the complementary filter for attitude estimation (i.e., the cut-off frequency of CF1). The attitude errors of SINS and CNS subsystems obtained in the static test are analyzed in time and frequency domain, and the analysis results are shown in Figure 3, Figure 4 and Figure 5 where $\phi_{x}$, $\phi_{y}$, and $\phi_{z}$ denote the pitch, roll, and yaw misalignment angles, respectively, the unit of which is arcsecond (″); t denotes the time axis, and its unit is second (s); $\left|\phi_{x}\right|$, $\left|\phi_{y}\right|$ and $\left|\phi_{z}\right|$ denote the amplitudes of the pitch, roll, and yaw error spectrums, the unit of which is arcsecond (″); f denotes the frequency axis, its unit is Hertz (Hz); $\sigma \left(\phi_{x}\right)$, $\sigma \left(\phi_{y}\right)$, and $\sigma \left(\phi_{z}\right)$ denote the standard deviations of attitude errors in SINS/CNS integration, and $f_{1}^{c}$ denotes the cut-off frequency of CF1. Figure 3 shows the time curves and amplitude spectrums of CNS attitude errors, of which the three subfigures on the left are the time curves, and three subfigures on the right are the amplitude spectrums. Figure 4 shows the time curves and amplitude spectrums of SINS attitude errors. Similarly, the three subfigures on the left are the time curves, and the three subfigures on the right are the amplitude spectrums. It can be seen from Figure 3 to Figure 4 that the attitude errors of CNS do not diverge and are less than 100″, while the time curves of attitude errors are characterized by disorderly jumping noise. According to the corresponding amplitude spectrums, the errors of CNS can be approximated as white noise. The attitude errors of SINS have a tendency to oscillate and diverge, which are close to $20^{\prime }$ in 3600 s. From the amplitude spectrums, it can be seen that the attitude errors of SINS are mainly distributed in the low frequency band of 0–1 Hz. In order to obtain the statistical relationship between the cut-off frequency $f_{1}^{c}$ and attitude estimation accuracy $\sigma \left(\phi_{i}\right)$, let $f_{1}^{c}$ take different values, and Monte-Carlo simulation is performed to record the corresponding values of $\sigma \left(\phi_{i}\right)$ (i=x,y,z) and $f_{1}^{c}$ every time. Selection of $f_{1}^{c}$ value points: the cut-off frequency value points are in the frequency range of 0 to 2.5 Hz, while the interval between two adjacent cut-off frequency points is taken as 0.03 Hz. In addition, the three frequency points of 0.001, 0.005, and 0.01 Hz are also tested. This means that the set of $f_{1}^{c}$ value points is ${\left[\begin{array}{cccc} 0.001 & 0.005 & 0.01 & \left[0.03:0.03:2.5\right] \end{array}\right]}^{T}\mathrm{Hz}$. According to the statistical results of Monte-Carlo simulation, the relationships between $f_{1}^{c}$ and $\sigma \left(\phi_{i}\right)$ (i=x,y,z) are obtained. As shown in Figure 5, the effect of cut-off frequency on the attitude error can be seen. The data pairs including the minimum values of $\sigma \left(\phi_{i}\right)$ (i=x,y,z) and corresponding $f_{1}^{c}$ are pitch (0.09 Hz, 0.2116’), roll (0.09 Hz, 0.2140’), and yaw (0.12 Hz, 0.2121’), which are marked with red asterisks(*) in Figure 5. It can be seen that the cut-off frequencies corresponding to the minimum values of three attitude errors are very close. In order to reduce the algorithmic complexity and computational load, the same cut-off frequency values are selected for the pitch, roll, and yaw estimations, i.e., $f_{1}^{c}=0.1\;\mathrm{Hz}$, $\omega_{1}^{c}=2\pi f_{1}^{c}=0.2\pi$.

In order to select the appropriate cut-off frequency of a complementary filter for velocity estimation (i.e., the cut-off frequency of CF2, $f_{2}^{c}$), the velocity errors of the SINS and GPS subsystems obtained in the static test are analyzed in the time and frequency domains, and the analysis results are shown in Figure 6, Figure 7 and Figure 8, where $\delta V_{E}$, $\delta V_{N}$, and $\delta V_{U}$ denote the velocity errors in the East, North, and Up directions, respectively, the unit of which is meters per second (m/s); t denotes the time axis, and its unit is second (s); $\left|\delta V_{E}\right|$, $\left|\delta V_{N}\right|$, and $\left|\delta V_{U}\right|$ denote the amplitude spectrums of velocity errors in the East, North, and Up directions, the unit of which is meters per second (m/s); f denotes the frequency axis, its unit is Hertz (Hz); $\sigma \left(\delta V_{E}\right)$, $\sigma \left(\delta V_{N}\right)$, and $\sigma \left(\delta V_{U}\right)$ denote the standard deviations of velocity errors in SINS/GPS integration, and $f_{2}^{c}$ denotes the cut-off frequency of CF2. Figure 6 shows the time curves and amplitude spectrums of GPS velocity errors, of which three subfigures on the left are the time curves, and three subfigures on the right are the amplitude spectrums. Figure 7 shows the time curves and amplitude spectrums of SINS velocity errors. Similarly, three subfigures on the left are the time curves, and three subfigures on the right are the amplitude spectrums. It can be seen from Figure 6 and Figure 7 that the velocity errors of GPS do not diverge and are all less than 0.5 m/s, the time curves of GPS velocity errors are characterized by disorderly jumping noise. According to the corresponding amplitude spectrums, the velocity errors of GPS can be approximated as Gaussian white noise. The velocity errors of SINS have a tendency to oscillate and diverge, which are close to 20 m/s in 3600 s. From the amplitude spectrum, it can be seen that the velocity errors of SINS are mainly distributed in the low frequency band of 0 to 0.5 Hz. In order to obtain the statistical relationship between the cut-off frequency value and velocity estimation accuracy, let the cut-off frequency $f_{2}^{c}$ take different values, and Monte-Carlo simulation is performed to record the values of $\sigma \left(\delta V_{i}\right)$ (i=E,N,U) and corresponding $f_{2}^{c}$. For Monte-Carlo simulation of CF2, the selection of $f_{2}^{c}$ value points is the same as that of $f_{1}^{c}$. According to the statistical results of Monte-Carlo simulation, the relationships between $f_{2}^{c}$ and $\sigma \left(\delta V_{i}\right)$ (i=E, N, U) are obtained, as shown in Figure 8, so the effect of cut-off frequency on the error of velocity estimation can be seen. The data pairs including the minimum values of $\sigma \left(\delta V_{i}\right)$ and corresponding $f_{2}^{c}$ are East (0.03 Hz,0.0413m/s), North (0.03 Hz,0.0408m/s), and Up (0.03 Hz,0.0381m/s), which are marked with red asterisks(*) in Figure 8. It can be seen that the cut-off frequencies corresponding to the minimum values of the three velocity errors are very close. Therefore, the same cut-off frequency value can be selected for velocity estimation; there, it is selected as $f_{2}^{c}=0.03\;\mathrm{Hz}$, $\omega_{2}^{c}=2\pi f_{2}^{c}=0.06\pi$.

Finally, the cut-off frequency is selected for position estimation (i.e., the cut-off frequency of CF3, $f_{3}^{c}$). The position errors of SINS and GPS subsystems obtained in the static test are analyzed in time and frequency domain, and the analysis results are shown in Figure 9, Figure 10 and Figure 11. Thereinto, δL, δλ, and δH denote the latitude, longitude, and altitude errors, respectively, the unit of which is meter (m); t denotes the time axis, and its unit is second (s); |δL|, |δλ|, and |δH| denote the amplitude spectrums of latitude, longitude, and altitude errors, the unit of which is meter (m); f denotes the frequency axis, its unit is Hertz (Hz); σ(δL), σ(δλ), and σ(δH) denote the standard deviations of position errors in SINS/GPS integration; and $f_{3}^{c}$ denotes the cut-off frequency of CF3. Figure 9 shows the time curves and amplitude spectrums of GPS position errors, of which three subfigures on the left are the time curves, and three subfigures on the right are the amplitude spectrums. Figure 10 shows the time curves and amplitude spectrums of SINS position errors. Similarly, the three subfigures on the left are the time curves, and the three subfigures on the right are the amplitude spectrums. It can be seen from Figure 9 and Figure 10 that the position errors of GPS do not diverge and are less than 30 m, while the time curves of the GPS position errors are characterized by disorderly jumping noise. According to the corresponding amplitude spectrums, the position errors of GPS can be approximated as Gaussian white noise. The position errors of SINS will oscillate and diverge, which are close to the order of magnitude of $2\times 10^{4}\;m$ at 3600 s. As seen from the amplitude spectrum, the position errors of SINS are mainly distributed in the low frequency band of 0 to 2 Hz. Similarly, in order to obtain a statistical relationship between the cut-off frequency and position estimation accuracy, let the cut-off frequency $f_{3}^{c}$ take different values, and Monte-Carlo simulation is performed to record the values of $\sigma \left(\delta P_{i}\right)$ (i=L,λ,H) and corresponding $f_{3}^{c}$; the selection of $f_{3}^{c}$ value points is the same as that of $f_{1}^{c}$. According to the statistical results of Monte-Carlo simulation, the relationships between $f_{3}^{c}$ and $\sigma \left(\delta P_{i}\right)$ (i=L,λ,H) are obtained. As shown in Figure 11, the effect of cut-off frequency on the position errors of SINS/GPS integration can be seen. The data pairs including the minimum values of $\sigma \left(\delta P_{i}\right)$ and corresponding $f_{3}^{c}$ are latitude (0.15 Hz,6.7436m), longitude (0.15 Hz,5.6282m), and altitude (0.09 Hz,6.2559m), which are marked with red asterisks(*) in Figure 11. The cut-off frequencies corresponding to the minimum values of the latitude, longitude, and altitude errors are very close. Based on the analysis above, the value of $f_{3}^{c}$ can be selected as $f_{3}^{c}=0.12\;\mathrm{Hz}$, $\omega_{3}^{c}=2\pi f_{3}^{c}=0.24\pi$.

The minimum standard deviations of navigation parameters and corresponding cut-off frequencies are summarized in Table 1. Finally, the values of $f_{1}^{c}$, $f_{2}^{c}$, and $f_{3}^{c}$ are determined as 0.1, 0.03, and 0.12 Hz, respectively.

## 5. Simulation and Experiment

The real-time data of an SINS/CNS/GPS integrated navigation system of an aerospace plane in flight are difficult to obtain. In this paper, the method of trajectory simulation combined with the error characteristics of a real sensor’s outputs is used to generate the simulation data of each navigation subsystem, and the semi-physical simulation of an SINS/CNS/GPS integrated navigation algorithm based on CF is performed to verify the validity. The semi-physical simulation experiment using this system has important practical significance for studying the characteristics of a SINS/CNS/GPS integrated navigation system of spacecraft under actual noise. In order to fully verify the priority of CF in real-time performance, an experimental test based on a real-time system was done.

### 5.1. Design and Implementation of Semi-Physical Simulation System

Combined with the application background of spacecraft, the semi-physical simulation platform of the SINS/CNS/GPS integrated navigation system is constructed, which consists of the hardware part (including IMU, GPS, CNS, navigation computer, etc.) and software part (including the trajectory simulation, integrated navigation algorithm, SINS updating algorithm, etc.). By modeling the sampling data of SINS, GPS, and CNS, the error characteristics of each navigation subsystem are obtained. According to the presupposed maneuver conditions, the required error-free data of navigation sensors are generated by using the method of trajectory simulation. According to the error characteristics of each subsystem, the real outputs of subsystems can be simulated by adding errors to the corresponding error-free data. Finally, the algorithm software of SINS and SINS/CNS/GPS integration are performed, and the navigation results are output.

**Figure 12:**
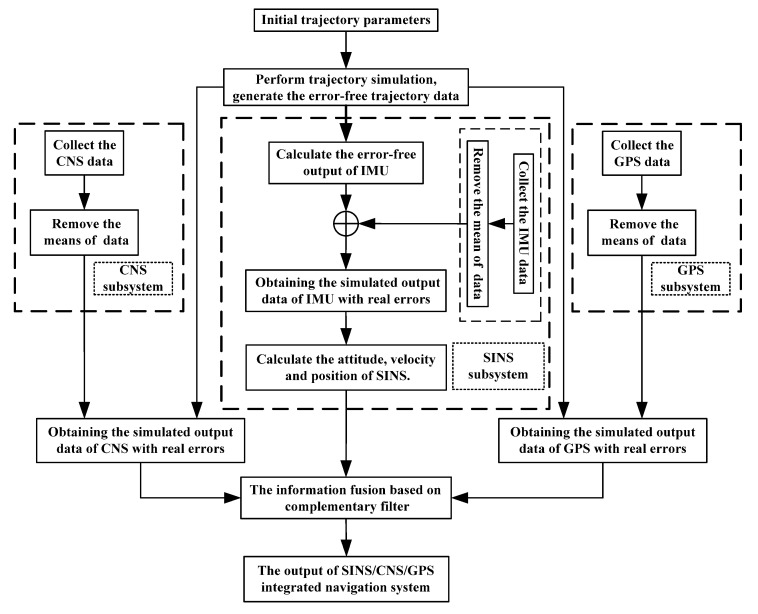
Flow chart of semi-physical simulation for SINS/CNS/GPS integrated navigation.

The flow chart of semi-physical simulation for an SINS/CNS/GPS integrated navigation system is shown in Figure 12. The process is as follows:

(1) Set the initial parameters of trajectory simulation, perform the trajectory simulation, and generate the error-free trajectory data (including the error-free attitude, velocity, and position of spacecraft.)
(2) According to the obtained trajectory data, calculate the error-free output of IMU. Collect the IMU data in a static test, and subtract the corresponding mean value from the IMU data to obtain the error data of inertial sensors. Then, add the error data to the error-free IMU data, thus obtaining the simulated output data of IMU with real errors. Then, calculate the attitude, velocity, and position of the SINS.
(3) The speed and position data of GPS in the static test are collected, and the means are subtracted from collected data to obtain the error data of GPS. Add the GPS’s error data to the error-free position/velocity data of spacecraft from trajectory simulation generated in step (1), thereby obtaining the simulated output data of GPS with real errors.
(4) Similarly, the error data of the CNS can be obtained by subtracting the true value of attitude from the collected off-line attitude data of CNS. Then, add the error data of the CNS to the error-free attitude data of the spacecraft from the trajectory simulation generated in step (1), thereby obtaining the simulated output data of CNS with real errors.
(5) By using the data from navigation subsystems generated in steps (2), (3), and (4), the information fusion based on complementary filter is performed to obtain the optimal estimations of attitude/velocity/ position parameters of an SINS/CNS/GPS integrated navigation system.

### 5.2. Simulation Conditions

Based on CF and FKF respectively, the simulations of SINS/CNS/GPS integrated navigation are performed using MATLAB software (MATLAB 2009a, MathWorks, Natick, MA, USA) with the simulation conditions as follows:

(1) Initial State of Aerospace Plane

The initial position (the longitude, latitude, and altitude in WGS84 frame) is $165.36^{o}\;W$, $34^{o}\;N$, and 406655.29 m; the initial velocity (including the velocity components in the East, North, and Up directions) is 2995.2 m/s, 6665.7 m/s, and 8.6 m/s; the initial attitude (including the pitch, roll, and azimuth angles) is $0.15^{{}^{\circ}}$, $0.1^{{}^{\circ}}$, and $67.3^{{}^{\circ}}$.

(2) Initial Errors of INS

The initial position errors (including the initial errors of longitude, latitude, and altitude) are 300m, 300m, and 300m. The initial attitude errors (including the initial errors of pitch, roll, and azimuth) are $10^{\prime }$, $10^{\prime }$, $10^{\prime }$. The initial velocity errors (the velocity errors in the East, North, and Up directions) are 1m/s, 1m/s, and 1m/s.

(3) IMU, GPS, and CNS Errors

Gyro errors: the random constant drift is $0.03^{{}^{\circ}}/h$ (1σ), and the angular random walk coefficient is $0.005^{{}^{\circ}}/\sqrt{h}$ (1σ). Accelerometer errors: the random constant bias is 30μg (1σ), and the velocity random walk coefficient is $5\mu g\sqrt{\mathrm{Hz}}\;(1\sigma )$. The sampling frequency of IMU is 200Hz.

GPS errors: the horizontal position error is 10m (1σ), the altitude error is 10m (1σ), and the velocity error is 0.1m/s (1σ). The sampling frequency of GPS is 1Hz.

CNS errors: the attitude error is $20^{\prime \prime }$ (1σ). The sampling frequency is 5 Hz.

(4) Parameter Setting of CF

The cut-off frequencies of complementary filters for attitude/velocity/position estimations are set as $f_{1}^{c}=0.1\;\mathrm{Hz}$, $f_{2}^{c}=0.03\;\mathrm{Hz}$, and $f_{3}^{c}=0.12\;\mathrm{Hz}$, respectively. The simulation time is 3600s.

### 5.3. Simulation Results

**Figure 13:**
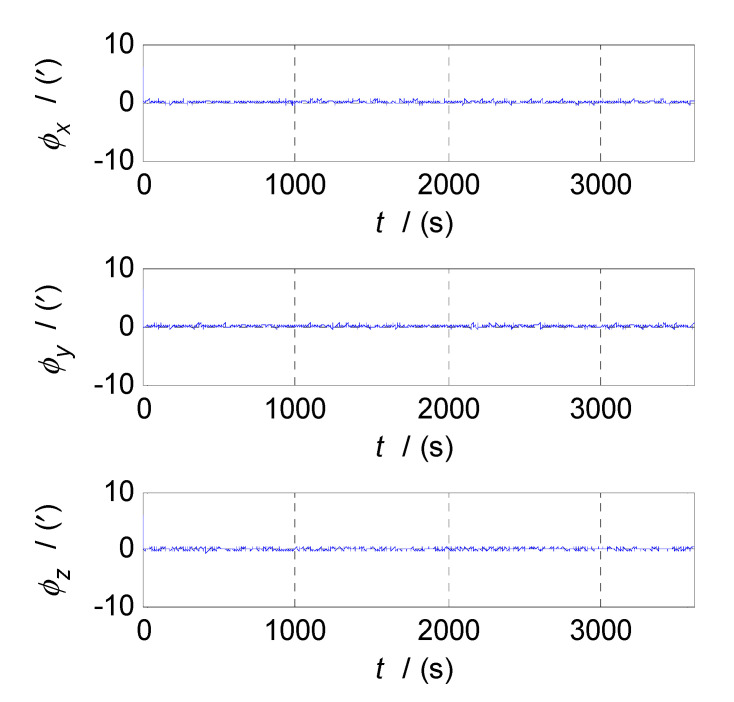
Attitude errors of SINS/CNS/GPS integration using complementary filter (CF).

**Figure 14:**
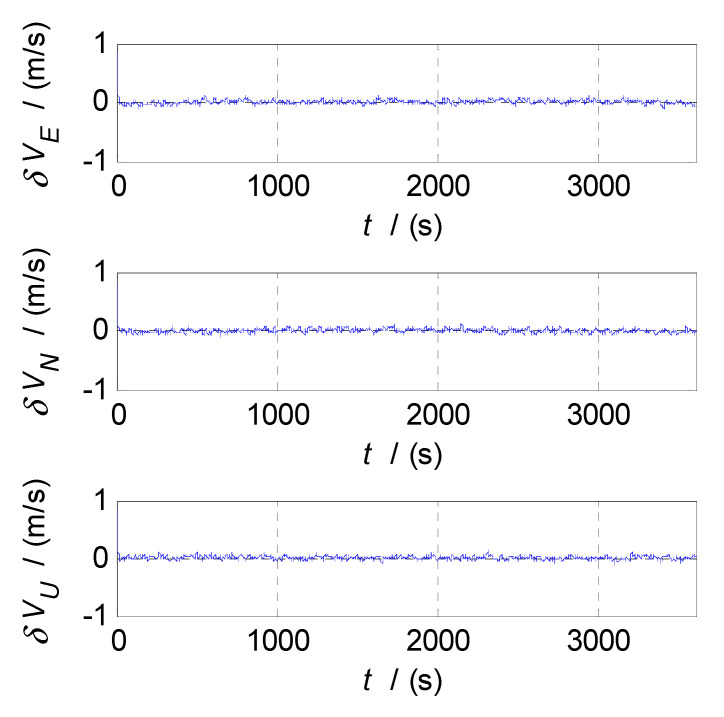
Velocity errors of SINS/CNS/GPS integration using CF.

**Figure 15:**
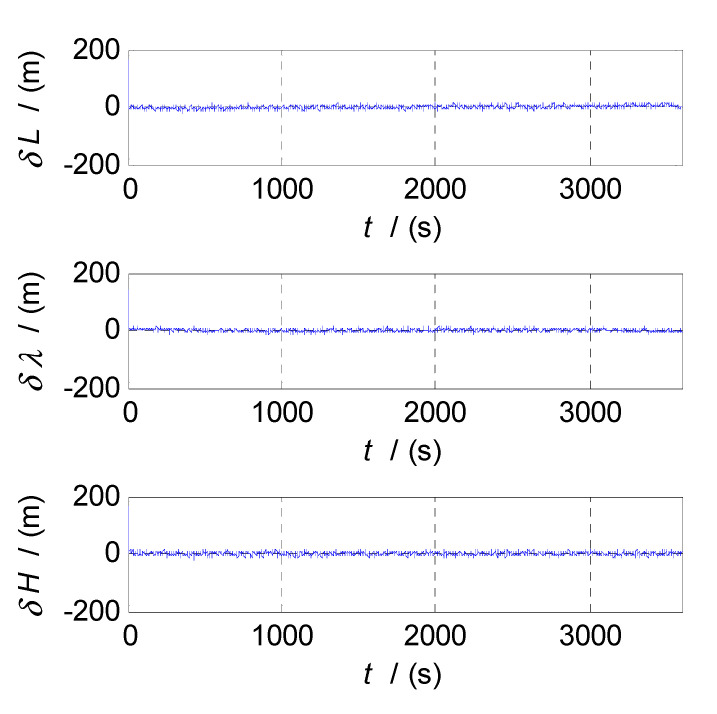
Position errors of SINS/CNS/GPS integration using CF.

The semi-physical simulation of the proposed CF-based SINS/CNS/GPS integrated navigation algorithm is performed, and the navigation errors obtained by comparing the integrated navigation results with the corresponding trajectory parameters generated by the trajectory generator are shown in Figure 13, Figure 14 and Figure 15. Figure 13, Figure 14 and Figure 15 show the time curves of attitude errors, velocity errors, and position errors, respectively. According to the statistics of errors, the standard deviations of pitch, roll, and azimuth errors are $0.2124^{\prime }$, $0.2137^{\prime }$, $0.2100^{\prime }$; the standard deviations of velocity errors in the East, North, and Up directions are 0.0426m/s, 0.0420m/s, and 0.0401m/s; the standard deviations of position errors in latitude, longitude, and altitude are 6.8851m, 5.5885m, and 6.4637m.

From Figure 13, Figure 14, Figure 15, when the CF processes of INS/CNS/GPS integration navigation start, the curves of attitude errors, velocity errors, and position errors converge quickly, and the noise components of navigation errors are basically filtered out. Throughout the process of simulation, the navigation parameters output by the CF-based SINS/CNS/GPS integrated navigation system remain convergent. The attitude errors are less than $0.7^{\prime }$, the velocity errors are less than 0.15 m/s, and the position errors are less than 10 m. The errors of navigation parameters are all within the acceptable range. So, the proposed method of CF-based information fusion can meet the requirements of long-term and high-precision navigation for an SINS/CNS/GPS integrated navigation system of an aerospace plane.

In order to further analyze the performance of the proposed algorithm, the semi-physical simulation of SINS/CNS/GPS integrated navigation algorithm based on FKF (federated Kalman filter) is carried out under the same simulation conditions. The estimation errors of CF are compared with that of FKF, as shown in Table 2. For both CF and FKF, the mean value of each navigation parameter error is one order of magnitude smaller than the standard deviation of the corresponding error. The means of errors are no longer listed in the table, and only the standard deviations of errors are compared and analyzed. The simulation results show that the statistical errors of the CF-based integrated navigation algorithm are close to that of the FKF-based integrated navigation algorithm. In terms of filtering accuracy, the estimation errors of the two filtering methods are in the same order of magnitude; however, in terms of the real-time and complexity, because FKF needs to perform a large number of high-order matrix operations, the computational complexity of FKF is much higher than that of CF, and the real-time performance of FKF is much poorer than that of CF. 

### 5.4. Analysis of Real-Time Performance

In order to investigate the real-time performance of the proposed CF-based information fusion algorithm for SINS/CNS/GPS integrated navigation, the average single-time elapsed times of the FKF and CF are recorded by using TIC and TOC functions of MATLAB. Denote the average single-time elapsed times of the FKF and CF as $t_{FKF}$ and $t_{CF}$, respectively. According to the recorded time, we have $t_{CF}=\;3.3836\times 10^{-5}s$ and $t_{FKF}=6.5180\times 10^{-4}s$. In order to eliminate the effect resulted from the difference of computer performances, the relative elapsed times of FKF and CF are defined as:

(40) $$\left\{ \begin{array}{l} t_{CF}^{R}=t_{CF}^{}/t_{FKF}\\ t_{FKF}^{R}=t_{FKF}^{}/t_{FKF}^{} \end{array}\right..$$

**Figure 16:**
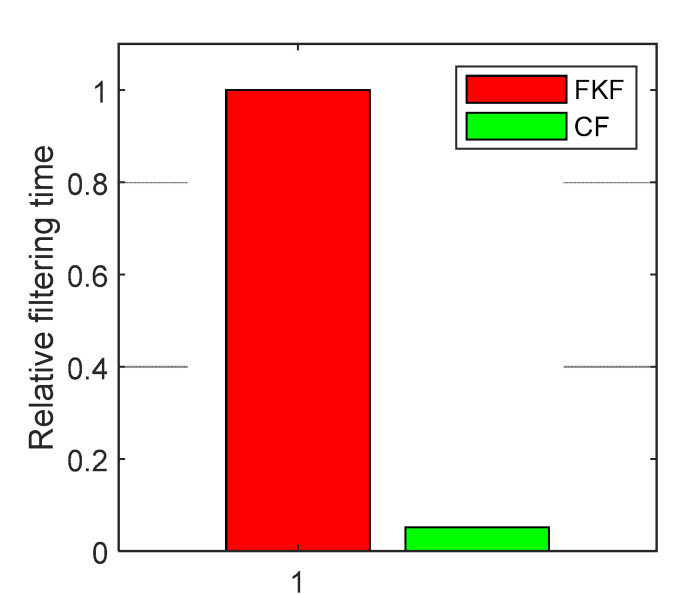
Relative time consumptions of FKF and CF.

Obviously, $t_{FKF}^{R}=1$, $t_{CF}^{R}=0.0519$. The relative elapsed times of FKF and CF are shown in Figure 16. From Figure 16, it can be seen that for the information fusion of SINS/CNS/GPS integrated navigation, the elapsed time of the CF is much shorter than that of the FKF, and the average single-time elapsed time of the CF is just 5.19% of the average single-time elapsed time of the FKF. The above simulation results demonstrate that the proposed CF-based method of information fusion for multi-sensor integrated navigation system successfully overcomes the drawback of heavy computational load of the FKF-based method. When used for SINS/CNS/GPS integrated navigation, the proposed CF-based method can save 94.81% computational time compared with the FKF-based method.

### 5.5. Experimental Test Based on Real-Time System

**Figure 17:**
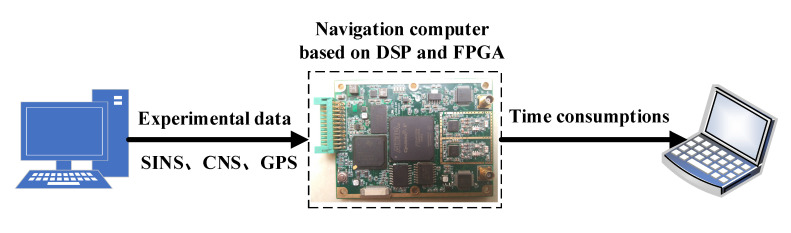
Data processing architecture based on DSP and FPGA chips for real-time system experiment.

In order to further verify the priorities of a proposed CF-based information fusion algorithm in terms of computational complexity and real-time performance, an experimental test based on a real-time system was conducted. The data processing architecture of a real-time system is shown in Figure 17, in which the navigation computer mainly includes Digital Signal Processor (DSP, Texas Instruments, TMS320C6748), Field Programmable Gate Array (FPGA, Altera, Cyclone V 5CEFA9F23I7N), Analog-to-Digital Converter (ADC, Analog Devices Inc., Norwood, MA, USA, AD9288), etc. The DSP chip is used to calculate the information fusion algorithms of SINS/CNS/GPS integrated navigation. The experimental process is as follows: Firstly, a frame of experimental data (including the data of SINS, CNS, and GPS) generated by semi-physical simulation is input into the DSP chip through the RS232/422 serial communication interface of the navigation computer. Subsequently, the two information fusion algorithms of SINS/CNS/GPS integrated navigation based on CF and FKF are carried out by the DSP chip, respectively. Eventually, the recorded time consumptions are output to a terminal display device.

The computational times of CF and FKF are shown in Table 3. It can be seen that the time consumptions of CF and FKF are $3.4795\times 10^{-6}$ s and $5.5088\times 10_{}^{-5}$ s, respectively. So, the timing performance of CF is better than that of FKF. There, the time consumption refers to the average time to process a frame of experimental data.

## 6. Conclusions

In this paper, a novel information fusion method based on complementary filter is proposed for the SINS/CNS/GPS integrated navigation system of an aerospace plane. The transformation algorithm of the CNS quaternion from the i frame to the n frame is designed, and the CF-based attitude/speed/position estimations are designed and implemented for SINS/CNS/GPS integrated navigation system, respectively. By utilizing the time-frequency domain analysis, the optimal cut-off frequencies of complementary filters are obtained. Compared with the FKF-based method, it can improve the real-time performance and computational efficiency of information fusion of spacecraft-borne multi-sensor integrated navigation. In order to verify the proposed CF-based information fusion algorithm, a semi-physical simulation experiment platform is designed, and the algorithm validity is verified by a semi-physical simulation experiment. The experiment results show that the CF-based integration method has the advantages of fast convergence speed, acceptable noise suppression, and acceptable navigation accuracy. By comparing the two methods of CF-based and FKF-based, it can be concluded that the accuracy of the CF-based method is close to that of the most commonly used FKF-based method; however, the CF has better real-time performance and lower computational complexity than FKF. For a SINS/CNS/GPS integrated navigation system of spacecraft, the elapsed time of CF is decreased to 5.19% of FKF, while the computational efficiency of CF is increased by 94.81%. The experiment results prove that the computational efficiency of information fusion of SINS/CNS/GPS integrated navigation is improved.

## Figures and Tables

**Table 1:** Statistics of minimum standard deviations and corresponding cut-off frequencies.

Errors	$\sigma_{min}^{}$	$f_{c}^{}\left(\mathrm{Hz}\right)$	$f_{c}^{}\left(\mathrm{Hz}\right)$
$\phi_{x}$ ${(}^{\prime })$	0.2116	0.0900	0.1
$\phi_{y}$ ${(}^{\prime })$	0.2140	0.0900	
$\phi_{z}$ ${(}^{\prime })$	0.2121	0.1200	
$\delta V_{E}^{}$ (m/s)	0.0413	0.0300	0.03
$\delta V_{N}^{}$ (m/s)	0.0408	0.0300	
$\delta V_{U}^{}$ (m/s)	0.0381	0.0300	
δL (m)	6.7436	0.1500	0.12
δλ (m)	5.6282	0.1500	
δH (m)	6.2559	0.0900	

**Table 2:** Comparison of estimation errors between CF and federated Kalman filter (FKF).

Errors (1σ)	CF	FKF	CNS	GPS
$\phi_{x}\;{(}^{\prime })$	0.2124	0.1442	0.3333	N/A
$\phi_{y}\;{(}^{\prime })$	0.2137	0.1538	0.3333	N/A
$\phi_{z}\;{(}^{\prime })$	0.2100	0.1449	0.3333	N/A
$\delta V_{E}^{}\;(m/s)$	0.0426	0.0275	N/A	0.1
$\delta V_{N}^{}\;(m/s)$	0.0420	0.0164	N/A	0.1
$\delta V_{U}^{}\;(m/s)$	0.0401	0.0168	N/A	0.1
δL (m)	6.8851	2.3852	N/A	10
δλ (m)	5.5885	2.3878	N/A	10
δH (m)	6.4637	3.9670	N/A	10

**Table 3:** Time consumptions of CF and FKF.

Filters	Average Time Consumption(s)
CF	$3.4795\times 10^{-6}$
FKF	$5.5088\times 10_{}^{-5}$

## References

[1] Ding J., Balakrishnan S.N. (2015). Intelligent Constrained Optimal Control of Aerospace Vehicles with Model Uncertainties. J. Guid. Control Dynam..

[2] Li J., Zhao J.H., Sha X.Q., Li F. The Rotation Modulation Inertial Navigation System for Blackout Area during Hypersonic Reentry. Proceedings of the International Symposium on Optical Communication and Optical Fiber Sensors.

[3] Yang J., Kong L.T., Zhang Z. Research on SINS in Application of Reentry Navigation for Lifting Reentry Vehicles. Proceedings of the IEEE Chinese Guidance, Navigation and Control Conference (CGNCC).

[4] Goodman J.L., Propst C.A. Operational Use of GPS Navigation for Space Shuttle Entry. Proceedings of the IEEE/ON Position, Location and Navigation Symposium.

[5] Mikrin E.A., Rocket S.P., Mikhailov M.V., Orlovskii I.V., Rozhkov S.N., Krasnopolskii I.A. (2019). Satellite Navigation of Lunar Orbiting Spacecraft and Objects on the Lunar Surface. Gyroscopy Navig..

[6] Gottzein E., Kuehl C., Filippi H., Barrios-Montalvo A., Krauss P.A., Heim J. Lion Navigator GPS/Galileo Receiver for Spacecraft Navigation. Proceedings of the 24th International Technical Meeting of the Satellite Division of the Institute of Navigation (ION GNSS), ION.

[7] Wang W.B., Fan G.C., Niu F., Xu C.D. Analysis and Comparison of Robust Least Squares Estimation Based on Multi-constellation Integrated Navigation. Proceedings of the 2nd IEEE International Conference on Computer and Communications (ICCC).

[8] Mikrin E.A., Mikhailov M.V., Orlovskii I.V., Rozhkov S.N., Semenov A.S., Krasnopolskii I.A. (2019). Circumlunar Spacecraft Navigation Using the Measurements from Global Navigation Satellite Systems GLONASS, GPS, Galileo and BeiDou. Gyroscopy Navig..

[9] Biswas S.K., Dempster A.G. GNSS-based Spacecraft Navigation in Elliptical and High Earth Orbits Using Single Propagation Unscented Kalman Filters. Proceedings of the 5th Indian Control Conference (ICC).

[10] Ma X., Ning X., Chen X., Liu J. (2019). Geometric Coplanar Constraints-Aided Autonomous Celestial Navigation for Spacecraft in Deep Space Exploration. IEEE Access.

[11] Zhu R., Pan X.G., Wang J.Q., Zhou H.Y. Autonomous Navigation Algorithm for Spacecraft Based on Sun-Earth-Moon Astronomical Information. Proceedings of the International Conference on Applied Mechanics, Mechatronics and Intelligent Systems (AMMIS).

[12] Chen K., Zhou J., Shen F.Q., Sun H.Y., Fan H. (2020). Hypersonic boost-glide vehicle strapdown inertial navigation system/global positioning system algorithm in a launch-centered Earth-fixed frame. Aerosp. Sci. Technol..

[13] Hu G.G., Gao B.B., Zhong Y.M., Ni L.Q., Gu C.F. (2019). Robust Unscented Kalman Filtering with Measurement Error Detection for Tightly Coupled INS/GNSS Integration in Hypersonic Vehicle Navigation. IEEE Access.

[14] Trigo G.F., Theil S., Vandersteen J., Bennani S., Roux C. (2019). Robust Tightly Coupled Hybrid Navigation for Space Transportation. J. Spacecr. Rockets.

[15] Wang X.M., Zhang H., Gao X.D. Overview of the INS/CNS integrated navigation technology. Proceedings of the 9th International Symposium on Advanced Optical Manufacturing and Testing Technologies (AOMATT)—Optoelectronic Materials and Devices for Sensing and Imaging.

[16] Gou B., Cheng Y.M. (2019). INS/CNS Integrated Navigation Based on Corrected Infrared Earth Measurement. IEEE Trans. Instrum. Meas..

[17] Wang R., Xiong Z., Liu J.Y., Shi L.J. (2016). A new tightly-coupled INS/CNS integrated navigation algorithm with weighted multi-stars observations. J. Aerosp. Eng..

[18] Zhao H., Xiong Z., Shi L., Yu F., Liu J. (2016). A robust filtering algorithm for integrated navigation system of aerospace vehicle in launch inertial coordinate. Aerosp. Sci. Technol..

[19] Xu F., Fang J.C. Research on the application of ensemble neural network in integrated navigation system. Proceedings of the 6th International Symposium on Instrumentation and Control Technology.

[20] Zhao G., Shao W., Chen K., Yan J. Study on UKF based Federal Integrated navigation for High Dynamic Aviation. Proceedings of the International Symposium on Photoelectronic Detection and Imaging 2011—Space Exploration Technologies and Applications.

[21] Li H.L., Wu D.W., Zhang B., Yang B.F., Zhao Y.H. A new method for integrated navigation of hypersonic cruising aircraft using non-Keplerian orbits. Proceedings of the International Conference on Automatic Control, Mechatronics and Industrial Engineering (ACMIE).

[22] Ma W.H., Xu Y.J., Bao Y., Yang B. Autonomous Integrated Navigation Plan for Space Transfer Vehicle. Proceedings of the 2nd International Symposium on Systems and Control in Aerospace and Astronautics.

[23] Ma W.H., Luo J.J., Wang M.M., Zhu Z.X. Development of Distributed Integrated Navigation Simulator for Autonomous Spacecraft. Proceedings of the IEEE International Conference on Mechatronics and Automation.

[24] Xu S.Q., Zhou H.Y., Wang J.Q., He Z.M., Wang D.Y. (2019). SINS/CNS/GNSS Integrated Navigation Based on an Improved Federated Sage-Husa Adaptive Filter. Sensors.

[25] Hu G.G., Gao S.S., Zhong Y.M., Gao B.B., Subic A. (2016). Modified federated Kalman filter for INS/GNSS/CNS integration. J. Aerosp. Eng..

[26] Hu G.G., Gao S.S., Zhong Y.M., Gao B.B., Subic A. (2016). Matrix weighted multisensor data fusion for INS/GNSS/CNS integration. J. Aerosp. Eng..

[27] Gao B.B., Hu G.G., Gao S.S., Zhong Y.M., Gu C.F. (2018). Multi-sensor Optimal Data Fusion for INS/GNSS/CNS Integration Based on Unscented Kalman Filter. Int. J. Control Autom..

[28] Gao B.B., Hu G.G., Gao S.S., Zhong Y.M., Gu C.F. (2018). Multi-Sensor Optimal Data Fusion Based on the Adaptive Fading Unscented Kalman Filter. Sensors.

[29] Thau F.E. (1973). Observing the state of nonlinear dynamic systems. Int. J. Control.

[30] Mercorelli P. A switching observer for sensorless control of an electromagnetic valve actuator for camless internal combustion engines. Proceedings of the 50th IEEE Conference on Decision and Control and European Control Conference.

[31] Rajamani R. (1998). Observers for lipschitz nonlinear systems. IEEE Trans. Autom. Control.

[32] Liu S.Q., Zhu R. (2018). A Complementary Filter Based on Multi-Sample Rotation Vector for Attitude Estimation. IEEE Sens. J..

[33] Michailidis M.G., Agha M., Rutherford M.J., Valavanis K.P. A Software in the Loop (SIL) Kalman and Complementary Filter Implementation on X-Plane for UAVs. Proceedings of the International Conference on Unmanned Aircraft Systems (ICUAS).

[34] Islam M.A., Saha S. Loosely Coupled GPS/INS Integrated Navigation System Based on Kalman Filter and Complementary Filter for Aircraft. Proceedings of the 2nd International Conference on Electrical & Electronic Engineering (ICEEE).

